# Goal Oriented Behavior With a Habit-Based Adaptive Sensorimotor Map Network

**DOI:** 10.3389/fnbot.2022.846693

**Published:** 2022-05-10

**Authors:** Felix M. G. Woolford, Matthew D. Egbert

**Affiliations:** Artificial Life and Minds Lab, School of Computer Science, University of Auckland, Auckland, New Zealand

**Keywords:** habit, sensorimotor contingencies, minimal cognition, robot controller, adaptive autonomy, enactivism

## Abstract

We present a description of an ASM-network, a new habit-based robot controller model consisting of a network of adaptive sensorimotor maps. This model draws upon recent theoretical developments in enactive cognition concerning habit and agency at the sensorimotor level. It aims to provide a platform for experimental investigation into the relationship between networked organizations of habits and cognitive behavior. It does this by combining (1) a basic mechanism of generating continuous motor activity as a function of historical sensorimotor trajectories with (2) an evaluative mechanism which reinforces or weakens those historical trajectories as a function of their support of a higher-order structure of higher-order sensorimotor coordinations. After describing the model, we then present the results of applying this model in the context of a well-known minimal cognition task involving object discrimination. In our version of this experiment, an individual robot is able to learn the task through a combination of exploration through random movements and repetition of historic trajectories which support the structure of a pre-given network of sensorimotor coordinations. The experimental results illustrate how, utilizing enactive principles, a robot can display recognizable learning behavior without explicit representational mechanisms or extraneous fitness variables. Instead, our model's behavior adapts according to the internal requirements of the action-generating mechanism itself.

## 1. Introduction

### 1.1. A Novel Habit-Based Controller

An enactive approach to AI and robotics requires us to take seriously the roots of autonomous agency and sense-making (Froese and Ziemke, 2009). To gain insight into the nature of intelligence, we cannot be content with mimicking the dynamics of intelligent behavior within the constraints of externally imposed norms. We must also ask *why* a system generates its own normative dimensions, how are they grounded in the material processes of the agent as a self-organizing system, and how do they relate to an intrinsically meaningful perspective on the world. These questions must motivate the design of our artificial models.

In recent years, a rich notion of habit as a core feature of cognition has been explored by theorists focussing on aspects of autonomy, sense-making, and anti-representationalism in enactivism (Barandiaran and Di Paolo, 2014; Egbert and Barandiaran, 2014; Barandiaran, 2017; Ramírez-Vizcaya and Froese, 2019; Hutto and Robertson, 2020). Of particular interest to us is a line of investigation concerning how habit serves as an approximation of a fundamental unit of the sensorimotor domain of cognitive life, analogous to the role of the autopoetic cell as foundational to the biological domain of life (Buhrmann et al., 2013; Buhrmann and Di Paolo, 2017; Di Paolo et al., 2017; Di Paolo, 2019). In this view, a habit is a precarious but self-maintaining structure of sensorimotor activity, one that sustains itself as an entity over time by continually reproducing the conditions of its own performance.

Aspects of this view have been investigated through a computational model called the *Iterant Deformable Sensorimotor Medium* (IDSM) (Egbert and Barandiaran, 2014; Egbert and Cañamero, 2014; Egbert, 2018; Woolford and Egbert, 2019; Zarco and Egbert, 2019). The IDSM is essentially a mapping between a sensorimotor state and a change in motor state which is mutated as the medium is imprinted with a history of trajectories through a *sensorimotor space*. When coupled to a robot the medium serves as a controller which drives a kind of similarity-based behavior, in which the robot is driven to repeat the motor activity that it produced when it was historically in a similar sensorimotor state. As a behavior is repeated more frequently it in turn sustains and reinforces its influence on the IDSM mapping. Taken together, this facilitates the development of self-maintaining habitual behavior. Beyond the IDSM, a handful of other AI/robotics-type works drawing upon enactive theory have explored habits through different computational mechanisms or used comparable similarity-based mechanisms without being explicitly concerned with habit (Mirza et al., 2006; Iizuka and Di Paolo, 2007; Bedia et al., 2019; Georgeon and Riegler, 2019). Nevertheless, the scope of computational models of the enactive notion of habit remains relatively under-developed considering the relevance of habit to broader development of enactive cognitive science.

A recent criticism of the line of investigations working with the IDSM and related models is that they remain too minimal to provide an effective model of intelligent behavior, and that our artificial agents must be capable of developing an increasingly complex network of habits (Ramírez-Vizcaya and Froese, 2020). One of our recent works attempted to step in this direction by exploring how maintaining and refining a network of habits supported goal-oriented behavior acquired through evolutionary processes (Woolford and Egbert, 2020). Here we aim to push further in the direction of enriching the space of available computational models which can be used to explore habit-based cognition. To this end we present a new robot controller model, an *Adaptive Sensorimotor Map Network* (ASM-network). Building upon the kind of processes introduced with the IDSM, the ASM-network adaptively regulates the behavior of the robot as it engages with its environment, so as to maintain the viability of a structural organization within the model. That internal structure is motivated by the hypothesized organization of a sensorimotor agent as a structure of self-maintaining sensorimotor regularities (Di Paolo et al., 2017). The first half of this article thus details relevant elements of sensorimotor theory and adaptive sensorimotor agency, and then describes how the model captures some of these principles.

After presenting the model, we present an investigation to demonstrate its practical capacity as a tool for modeling cognition behavior. We investigate how a robot can solve a minimal cognition task previously investigated using evolutionary robotics methods (Beer, 1996). Evolutionary robotics methods have yielded invaluable developments in embodied theories of cognition through the analysis of the dynamics of adaptive behavior (Beer, 2008; Vargas et al., 2014). However, they have a critical limitation as an approach to investigating normativity and agency in an enactive sense, in that the viability constraints which the adaptive behavior maintains are externally imposed and have no meaningful correlation with the behavioral dynamics of the system. Barandiaran describes this as “the problem of dissociation between norm-establishing and norm-following processes” (Beer, 1997; Barandiaran and Egbert, 2014). Our investigation demonstrates that a system which attempts to reconcile these processes can still be used to investigate the same kinds of adaptive dynamics.

### 1.2. Sensorimotor Contingency Theory

Sensorimotor Contingency Theory is an attempt to account for the existence and quality of perceptual experience without appeals to notions of internal representation and other computational explanations (O'Regan and Noë, 2001; Noe, 2004). According to O'Regan and Noë's formulation of the theory, regularities in the relationship between movement and sensorimotor stimulation, and the “mastery” of such regularities, can explain an agent's phenomenal experience of perception as a result of their embodied activity. An archetypal example of the explanation provided by the theory is that of how the quality of “softness” is experienced. When a person squeezes a soft object such as a sponge, there is a particular contingent relationship between movements in the hand yielding a particular amount of pressure on the nerves in the finger tips. When squeezing a harder object such as a stone, the same muscle movements would coincide with a greater intensity of pressure on the fingertips. In mastering the laws of these relationships between motion and sensation the agent brings forth the experiences of softness and firmness, and the distinction between them. As O'Regan puts it, the experience of softness/firmness is a quality of the interaction in time between the body and the object, not an essential property of the object or “inside” the brain (O'Regan, 2011).

The theory's emphasis on perception as a process of active agent-environment interaction resonates with the enactive approach to cognition, especially with regard to the notion of sense-making. However the exact nature of sensorimotor contingencies and the notion of mastery in particular has proven challenging to reconcile with other aspects of enactivist thought. One challenge is the original formulation's apparent acceptance of cognitive representationalism to account for mastery (Hutto and Myin, 2012). Another is the question of how and why an autonomous agent would develop mastery of contingencies that are meaningful for that agent. Recently Di Paolo, Buhrmann, and Barandiaran provided a formalization of sensorimotor contingencies in terms of dynamical systems theory (Buhrmann et al., 2013). As part of this formalization, they defined four categories of sensorimotor contingencies, which describe different levels of the relationship between sensorimotor dynamics and the experience of the agent:

1. **Sensorimotor (SM) Environment**, the set of all regularities in the way that actions may affect perceptions for a given body in a given environment, e.g., between eye movements and retinal stimulations, without regard for the agent's internal dynamics involved in performing those actions.
2. **Sensorimotor Habitat** regular trajectories within the sensorimotor environment associated with a particular agent's way of being. In other words, the time-extended regularities involved in the loop of both action affecting perception *and* perception affecting action, given the specific internal properties of the acting agent.
3. **Sensorimotor Coordination**, a clustering of regularities within the sensorimotor habitat associated with the fulfillment of a particular goal of an agent.
4. **Sensorimotor Scheme**, an organization of coordinations associated with a particular normative framework and modulated according to that framework.

These categories clarify the distinction between (1) contingencies considered in more mechanical or statistical terms relating to the coupling between body and environment, and (2) contingencies as related to the experience of the agent in terms of its needs and expectations. We will briefly expand upon the details of these categories which are most relevant to this investigation. We are mostly concerned here with what it means for sensorimotor coordinations to be organized in relation to *goals* and *norms*.

**Figure 1:**
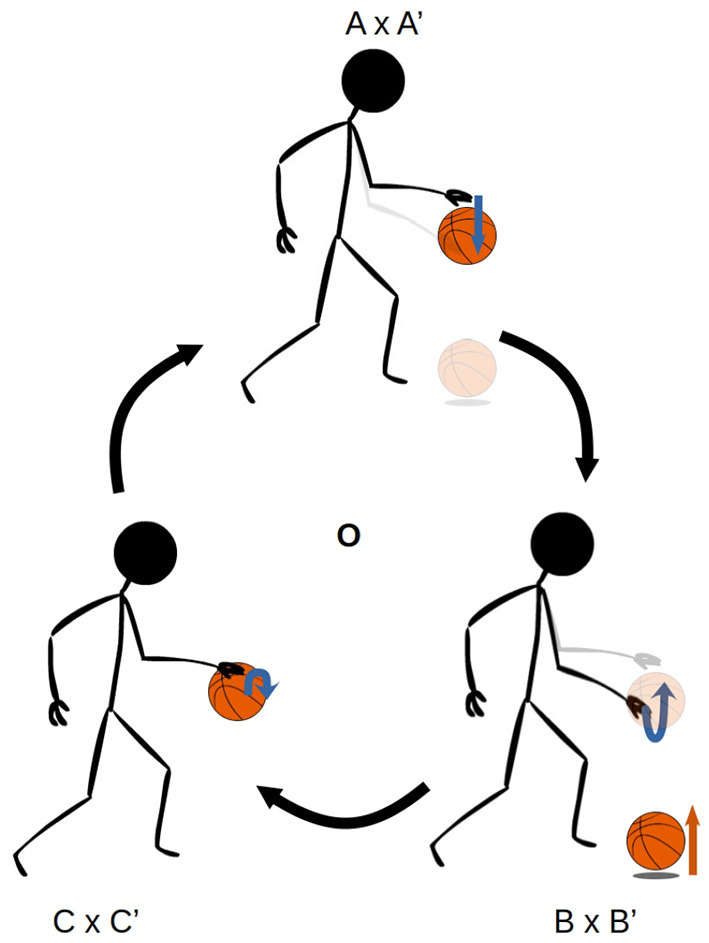
The sensorimotor scheme **O**, associated with the behavior of bouncing a basketball. The scheme consists of the cyclical organization of the three coordinations, A × A' → B × B' → C × C' → A × A', where the arrows indicate the transitional structure between these coordinations. The A × A' notation refers to the simultaneous realization of the agent-side sensorimotor support structure A, and the environment-side response structure A'.

**Figure 2:**
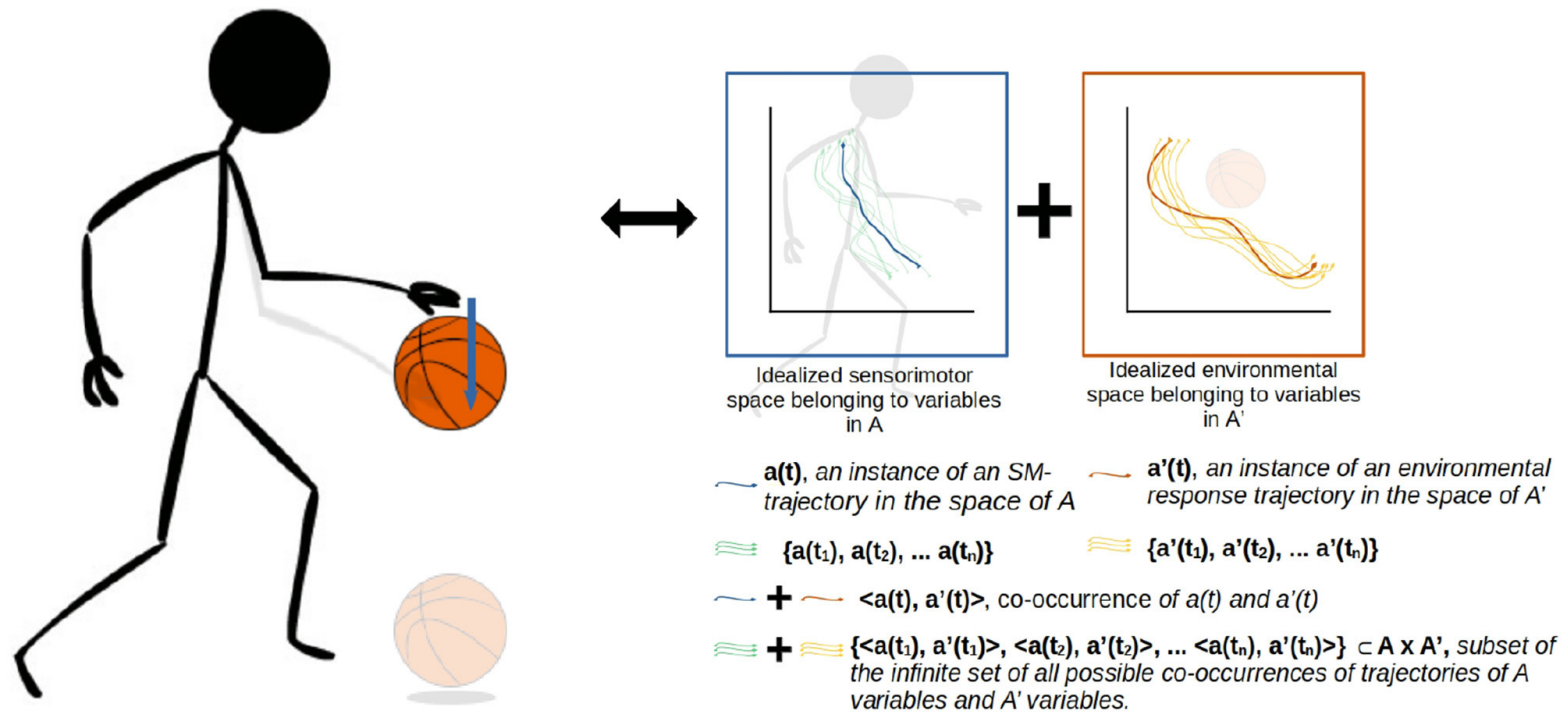
A more detailed visualization of the A × A' coordination from the previous figure. The coordination captures not just the agent's actions (A) and sensations but also the environmental processes that happen concurrently (A'). The state spaces represent theoretical projections of the state spaces of the relevant variables on the agent-side and environment-side of the engagement. The trajectories are not just co-occurring but also circularly causal, with the sensorimotor trajectory being a function of both internal (e.g., neuromuscular) dynamics and the environmental impacts on the agent's body, and similarly the environmental trajectory being a function of both the agent's actions and environmental processes such as gravity acting upon the ball.

Figures 1, 2 illustrate the way in which the bodily and environmental aspects of bouncing a basketball relate to the ideas of sensorimotor coordination and sensorimotor schemes. We can consider this as a **scheme** composed of three **coordinations**: Pushing the basketball toward the ground; preparing to receive the ball as it bounces on the ground; and receiving the ball as it returns to the hand. Each of these coordinations describes a particular class of embodied dynamics, all associated with a particular aspect of the basketball bouncing process. Assuming the scheme is stable, then each particular instance of enacting this scheme will follow the same sequence of coordinated acts, with each instance of a coordination varying in its precise dynamics but reliably establishing the enabling conditions for an instance of the next coordination. The processes involved in these transitions are honed over time with respect to various normative dimensions associated with bouncing a basketball effectively and efficiently.

A crucial emphasis of this formalization is that these regularities are not just concerned with the agent's brain and body, but involve the entirety of the brain-body-world system. The regularities associated with the performance of this scheme encompass both the positioning and readiness of the agent's body, and the position of the ball in relation to the body. Figure 2 illustrates how these regularities form a sensorimotor-coordination. Each coordination encompasses co-occurring regularities in the dynamics of both the agent (i.e., the actions and sensations associated with pushing the ball downwards, in the case of A here) and the environment (i.e., the position of the ball in space and its physical attributes, in the case of A'), within a specific temporal context with respect to several other coordinations. In other words, every instance of a particular coordination is a trajectory through a space of sensorimotor states (sensorimotor space), over which relevant state variables are transformed from one particular set of enabling conditions to another set, and a coordination structure ultimately is composed of the infinite set of possible variations on these trajectories.

In this example, we have outlined what an organization of sensorimotor contingencies might look like with respect to a particular activity, but not how or why such an organization would develop. Exactly whose goals and norms are we referring to when we say a scheme is associated with a particular normative framework, and where do those norms come from?

### 1.3. Sensorimotor Agency

Di Paolo et al. (2017) integrated their formalization of SMCs with a proposal for an account of cognition in which a complex embodied agent, such as a human, is not reducible to just its biological processes, but rather consists of many autonomous processes in deeply interwoven but ultimately irreducible biological, behavioral, and social domains. These processes and the relations between them ultimately ground the goals and norms which are relevant to the higher-level categories of sensorimotor contingencies. The core of their proposal is the idea that an organization of sensorimotor contingencies can manifest the necessary and sufficient properties to possess its own form of agency. Such an organization is proposed to constitute behavioral domain's analog to the notion of the cellular organism as biological agent. The short version of the definition of an agent that underlies the proposal is:

An autonomous system capable of adaptively regulating its coupling with the environment according to the norms established by its own viability condition (Barandiaran et al., 2009).

In the case of a sensorimotor agent, this system is a self-individuating, self-sustaining organization of activity which emerges within the dynamics of a brain-body-environment system, an entity composed of interacting sensorimotor schemes. This interaction refers to the relations between sensorimotor schemes in time—the way in which the performance of one scheme can regularly support, inhibit, or require the performance of other activities. At a high level we can think of each of these schemes as the regularities concerning a particular embodied activity: drinking from a cup, walking, reaching for a phone. Crucially, these are regularities which emerge not just in the dynamics of the internal process of the agent, but over the entire coupled system comprising the physical properties of the world, the agent's body, and the agent's neurological and physiological dynamics. A structure of interrelated activities can be understood as constituting its own kind of entity in the sensorimotor domain. Such a entity would comprise the entirety of the activities involved in a particular embodied agent's mode of being. The self-individuation of this structure refers to the way in which the stability of this structure is established through the very processes of activity that constitute it. These processes establish an operational closure of all of those activities which stabilize support for other activities within this structure, and in turn depend on the support of other activities in the structure. This process of self-individuation grounds a dimension of normativity related to the continuation of the activities which constitute the sensorimotor agent, as well as to the integrity of the structural relationships between activities. Actions and environmental structures may take on meaning of being more or less good or bad depending on how they support or disrupt that process. These elements may be irrelevant or even in direct opposition to the agent's viability at another level, such as the biological. If the dynamics of the brain-body-environment coupling are such that the behavior of the agent may change and develop to maintain its sensorimotor organization according to these norms, then we have an autonomous structure at the sensorimotor level which adaptively regulates its engagement with its environment, thus fulfilling the criteria of an organization that possesses its own form of agency.

This theory of sensorimotor agency has the potential to explain how and why an agent develops the sensorimotor mastery necessary to ground its phenomenal experience of the world, and to explain how complex behaviors and skills can take on “a life of their own,” apparently divorced from any role in maintaining the viability of the biological agent engaged in those behaviors. Clearly though, the idea of an agent constituted by its own acts presents a challenging conceptual puzzle (Di Paolo et al., 2017, Chapter 6). Artificial models have a key role to play in both clarifying and developing this and associated theories. Much of this work to date has focussed on the notion of *habit*, which provides a useful “first approximation” (Egbert and Barandiaran, 2014) of a minimal kind of self-sustaining sensorimotor entity. A habit may be conceived of as a dissipative structure of activity which depends upon its own continual re-performance for stability. In the context of the formalization of sensorimotor contingency categories, the structure of a minimal habit is akin to a single, circular scheme in which a series of coordinations ultimately reproduce the conditions for their own re-enactment. This structure grounds a single normative dimension concerning that continuing cycle of reproducing enabling conditions. Although the notion of habit—especially a single habit in isolation—does not capture the full richness of sensorimotor agency (Di Paolo et al., 2017, p. 146–154), it provides a starting point for investigation.

This brings us to our own work. Our aim is to build upon previous models that have been used to investigate this kind of enactive notion of habit, moving a step closer to the idea of sensorimotor agency proper. In particular our model aims to investigate the notion of habit more directly in terms of those categories of sensorimotor contingency, by explicitly incorporating properties of sensorimotor structure and dynamics which support the maintenance of that structure's viability in the face of environmental disruptions and obstacles. We now present a description of this model.

## 2. Model

### 2.1. An Overview of the ASM-Network Model

In the simplest description, the ASM-network model is a robot controller which generates motor commands for a robot based on the relationship between its current sensorimotor state and its history of sensorimotor trajectories. It consists of a network of Adaptive Sensorimotor Map units (ASM-units). The general design of each unit is similar to an earlier model, the Iterant Deformable Sensorimotor Medium (IDSM) (Egbert and Barandiaran, 2014; Egbert and Cañamero, 2014), while the mechanisms involved in organizing these units as a network are based on our previous Sensorimotor Sequence Reiterator model (Woolford and Egbert, 2020). Both of those models, and this one, may be considered as belonging to a family of habit-based robot controllers. These models are similar in two primary ways: Firstly, they are all specifically concerned with a sensorimotor level of abstraction (i.e., leaving aside lower level neural and physiological dynamics). Secondly, when coupled to the motors and sensors of an embodied robot as a controller, they serve to encourage the repetition and reinforcement of the robot's historical behaviors. The ASM-network is unique among these controllers in that it monitors the way in which new performances affect the stability of historically established behaviors, and *adaptively* modulates its own dynamics in the direction of maintaining the viability of those behaviors. Additionally, the processes of the model are organized analogously to the organization of sensorimotor contingencies in an autonomous sensorimotor entity as we described in the previous section.

**Figure 3:**
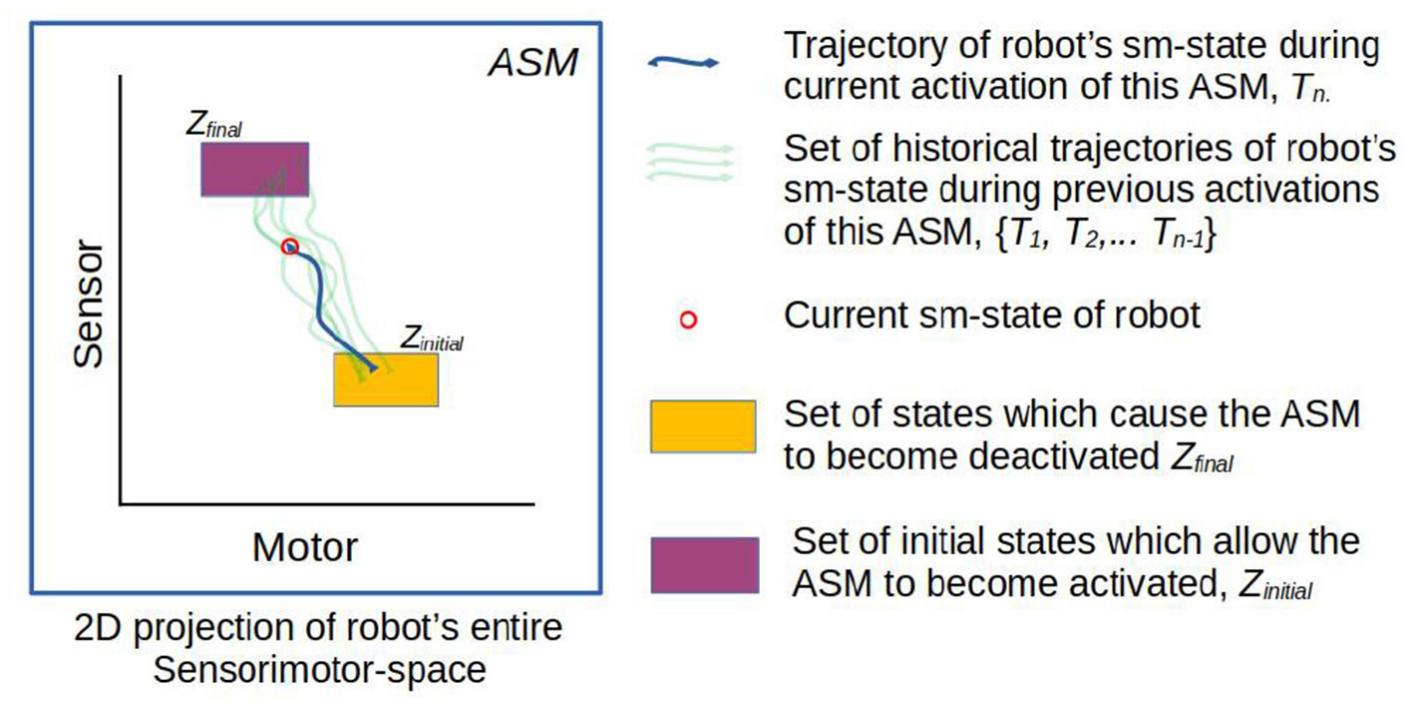
Simplified visualization of an ASM-unit in operation. The model essentially stores a number of historical trajectories in this space which have passed from the within range initial state to the range of final states, and utilizes information about those trajectories to generate motor activity for the robot in its current state.

**Figure 4:**
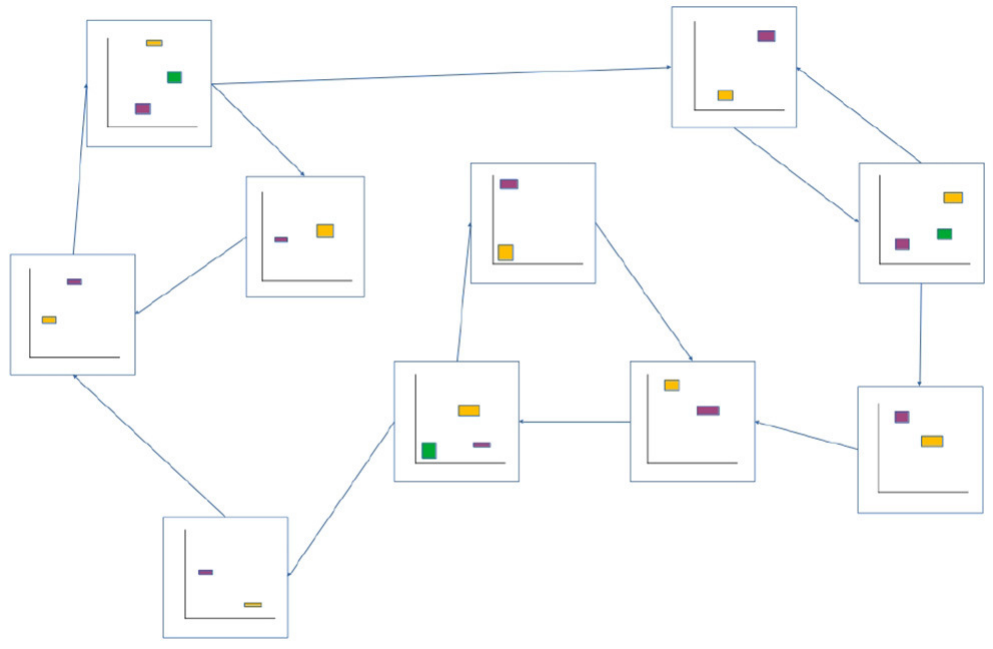
Example of an ASM-network consisting of 10 ASM-units. Like in Figure 3, ASM-units are represented by two-dimensional projections of sensorimotor-spaces. Arrows indicate that activation will transition from one ASM-unit to another when the controlled robot is in an appropriate sensorimotor-state. Note that the yellow initial-state regions of each ASM-unit corresponds in space with a purple or green final-state region from a preceding ASM-unit.

**Figure 5:**
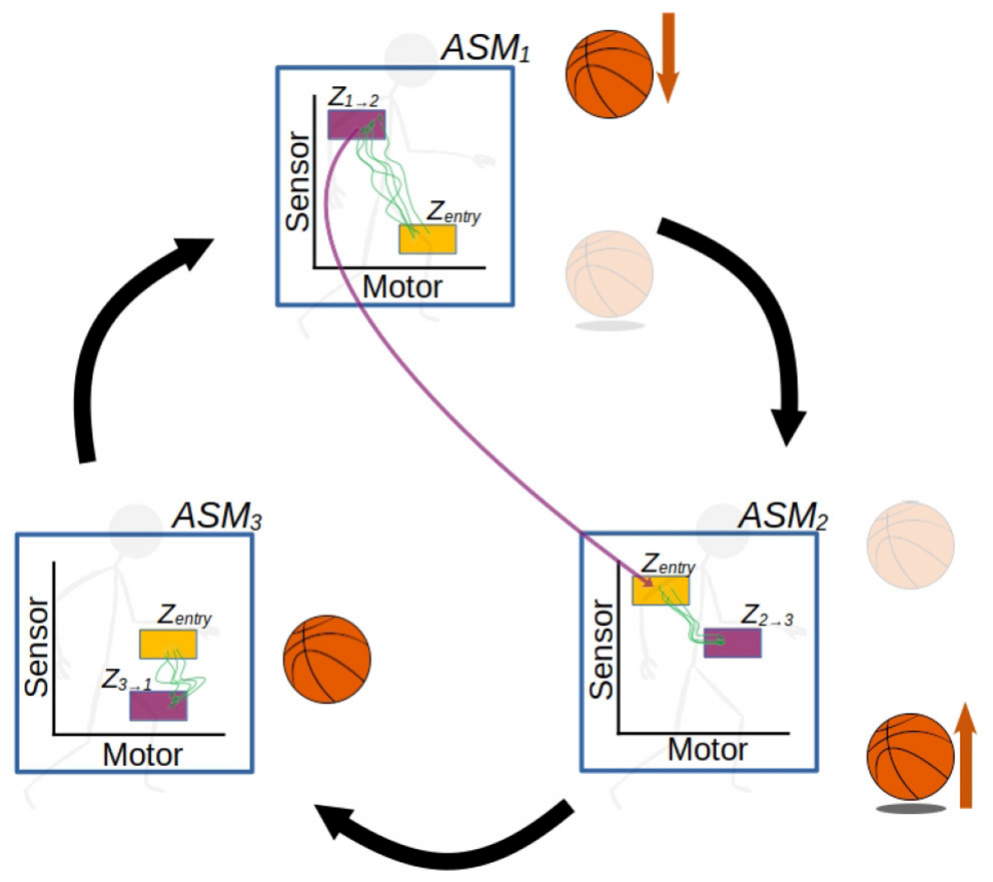
The activity of an ASM-network with three ASM-units, overlaying a visualization of the robot engaged in the ball-bouncing scheme. Each ASM-unit simulates a particular sequential component of the dynamics involved in the robot's side of the coupling. Each activation of the ASM-unit yields the necessary state for the activation of the next ASM-unit in the sequence. The progression of activations through the network mirrors the temporal arrangement of coordinations in a scheme.

Figures 3, 4 illustrate the basic elements of the ASM-network model. In operation, only one ASM-unit is “active” at any one time, and that unit is responsible for governing the changes in motor activity of the robot. This state of activation traverses the network over time. As a rough approximation, we may think of an individual ASM-unit as being associated with the agent dynamics associated with a single sensorimotor coordination structure, and a collection of these coordinations in a network as being associated with a sensorimotor scheme. Figure 5 illustrates a hypothetical relationship between our basketball-bouncing sensorimotor scheme and an instantiated ASM-network model in the context of a robot, controlled by an ASM-network, which is able to successfully enact that sensorimotor scheme. The model components illustrated there will become clear as we discuss further.

We now discuss the model in three parts: Firstly, we explain model at the level of individual ASM-units, and then at the network level. Finally, we will explain how these two levels interact to adaptively maintain stable behavior. Symbols used in the following sections are summarized in Table 1.

**Table 1:** Symbols for model parameters and components.

**Symbol**	**Value for section 3**	**Description**
τ	0.1 s	Period between node-creation events in an ASM-unit
*N*_*max*_	8,000	Maximum number of nodes in a single ASM-unit before nodes begin to be replaced
*w*	1.5	Scales the relative importance of *P* and *V* comparisons in similarity metric
*t*_*g*_	8 s	Period of activation for an ASM-unit before non-historical transitions may occur
*t*_*h*_	16 s	Maximum period of activation for an ASM-unit
*d*_*cutoff*_	0.2 sm-space units	Maximum distance in SM-space for candidate parent nodes
*N*	〈*P, V*, Δ*m, C, Z, A*〉	ASM-unit node
*P*	8-dimensional vector	Position in SM-space of a node
*V*	8-dimensional vector	Displacement of node's position from previously created node's position
Δ*m*	1-dimensional vector	Change-in-motor-state generated at node-creation
*C*	label	Node class label, inherited from parent node
*Z*	label	Label of the transition condition which terminates the activation of an ASM-unit
*A*	0 or 1	Flag which marks a node as reinforced or inhibited

*Described values for the parameters and dimensionality of vectors are those used in the investigation in Section 3 but are only indicative of a suitable order of magnitude for the general case*.

### 2.2. ASM Unit-Level Architecture

All ASM-units shares the same functional properties, and are essentially self-contained in their operation in most cases. Therefore, we can present most of the model in terms of a single ASM-unit in isolation from the rest of the network. Figure 3 illustrates the basic elements of an ASM-unit graphically. Readers familiar with the IDSM will recognize several core similarities in the ASM-unit's architecture.

A key concept at the heart of an ASM-unit is the notion of the *sensorimotor space*, the construct of all possible values of *all* sensor and motor variables of the controlled robot, which are each treated as bounded scalars. Conceptually we may think of these values as representing the full range of movements and sensations accessible to the robot. At any moment, the robot's sensorimotor state is the value of all of those sensors and motors:


(1) $$\begin{array}{l} \text{sm}\left(t\right)=\left[\begin{array}{c} m_{1}\left(t\right)\\ \vdots \\ m_{n}\left(t\right)\\ s_{1}\left(t\right)\\ \vdots \\ s_{n}\left(t\right) \end{array}\right] \end{array}$$

The ASM-unit essentially operates in terms of comparing the current sensorimotor state to historical states in terms of their position in sensorimotor space. In an example in the context of the basketball-bouncing scheme, we may think of it comparing a particular movement and sensation of the arm to other historical movements and sensations.

The ASM-unit also gives primacy to the concept of the time-extended trajectory of the robot's sensorimotor state through sensorimotor space. In the context of each ASM-unit, which is only active for a finite segment of time, we specifically use the term *sensorimotor trajectory* to refer to discrete segments of the robot's trajectory through the space, beginning at the time of the ASM-unit's activation and ending at the time of its termination. The ASM-unit therefore has a collection of historical sensorimotor trajectories, based on how many times it has been activated. Figure 3 illustrates a collection of five historical sensorimotor trajectories, suggesting that it the ASM-unit is currently in its sixth activation. As per the basketball example, we may think of each of these sensorimotor trajectories as instances of the robot's sensorimotor activity as it was going through a particular performance of a particular act, e.g., of pushing the ball.

An ASM-unit is designed so that it causes historical sensorimotor trajectories to be repeated, by dynamically generating a sensorimotor-state to change-in-motor-state map, *f*(**sm**) = ṁ, based on those historical sensorimotor trajectories. The dynamics of the model are precarious in that information of historical trajectories is lost over time, so for a particular behavior to be sustained over the long term it must regularly recur. However, repeating historical trajectories is not as simple as merely repeating historical motor actions. The time-extended evolution of the sensorimotor state may be separated into the evolution of the motor states and evolution of the sensor states:


(2) $$\begin{array}{l} \dot{\text{sm}}=ṁ+ṡ=\left[\begin{array}{c} \dot{m_{1}}\\ \vdots \\ \dot{m_{n}}\\ 0\\ \vdots \\ 0 \end{array}\right]+\left[\begin{array}{c} 0\\ \vdots \\ 0\\ \dot{s_{1}}\\ \vdots \\ \dot{s_{n}} \end{array}\right] \end{array}$$

(3) $$\begin{array}{l} \begin{array}{c} ṁ=f\left(\text{sm}\right)\\ ṡ=g\left(m,\text{e}\right) \end{array} \end{array}$$

Where, **e** is a vector representing the environmental state (i.e., properties of the world and the robot's position in it). Ultimately the ASM-unit is only responsible for generating *f*(**sm**) (see later, Equations 5–7), but has no direct influence on *g*(*m*, **e**). In other words, the ASM-unit is designed to reproduce historical sensorimotor trajectories, but it only has direct control over the change in state in a subset of the relevant dimensions. The same motor action in two different contexts may yield different sensorimotor trajectories depending on the environmental state. This produces a tension which causes only certain behaviors to be stable—those in which the repetition of certain sensorimotor states is concurrent with the repetition of certain environmental states. In our basketball example, this means that regular movements and sensations are only stable if the physical properties of the ball bouncing off the ground are also regular. This challenge relates to the concurrence of agent-side and environment-side dynamics in a sensorimotor coordination as illustrated in Figure 2.

In any non-trivial system, natural variations in the environmental state will mean that exact repetitions of historical trajectories are not possible, and thus the ASM-unit needs a mechanism for comparing the relative similarity of the current state to historical states. Thus, the influence of particular trajectories through sensorimotor space propagates over the entire state space, such that historical change-in-motor-state commands are adjusted for the current context. From a design perspective, the functional effectiveness of these comparisons and adjustments (i.e., the comparisons are accurate and the adjustments suitable) are critical to the ASM-unit's ability to repeat historical behaviors.

This brings us to the two operational mechanisms of an ASM-unit, (1) storing information about historical sensorimotor trajectories, and (2) using that information to generate change-in-motor-state commands. Sensorimotor trajectories are sampled at discrete intervals and stored by the ASM-unit as sequences of nodes, each representing the sensorimotor state of the robot at the moment of sampling. When determining a change-in-motor-state command, the ASM-unit compares the current SM-state to these stored nodes, and generates an output based on the state of the most similar stored node. The remainder of this subsection will explain the details of these two basic mechanisms.

#### 2.2.1. Node Creation

**Figure 6:**
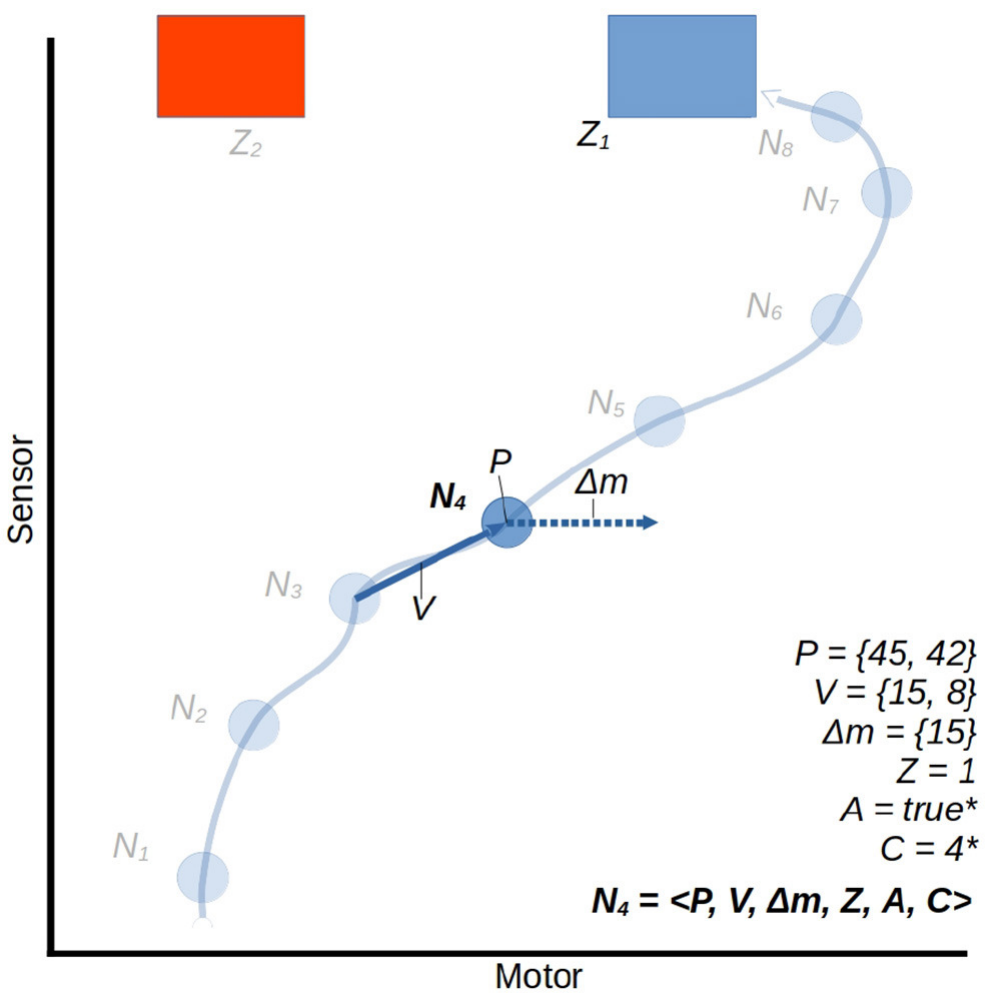
A trajectory through a 2D sensorimotor space, represented in an ASM-unit with eight nodes. We use the node *N*_4_ as an arbitrary example, all others share the same properties. For illustrative purposes we treat the motor and sensor dimensions abstractly as continuous ranges between 0 and 100. Note that the component *A* = 1 is determined in the context of subsequent activations of other ASM-units. This is explained in Section 2.4.

The controller is applied in a simulation of a continuous-time system, using the Euler method to approximate continuous dynamics, with a time step of size 0.01. At regular intervals of τ time units (τ = 0.1*s*) a node is created to store the current state of the robot, and a vector Δm is generated which determines the rate of change to the robot's motor state over the next τ interval. The structure of a node is illustrated in Figure 6. Upon creation, each node stores the following information:

1. The current sensorimotor state of the robot, which we regard as the node's position in sensorimotor space *P*,
2. The vector *V* from the position of the previously created node to the position of the current node,
3. The vector Δm for the intended change in motor state determined during node creation. The process of generating Δm is discussed shortly.
4. An identifying class label for the node, *C*, which is inherited from the most similar historical node (parent node) and will propagate to future similar nodes. This too is discussed further shortly.

There are also data stored in each node that relate to how the ASM-unit is exited. When the activation of an ASM-unit ends, all of those nodes which were created during that activation are modified to include:

5. An identifying label, *Z*, of the exit region that caused the activation to transition to another ASM-unit.

Finally, when the activation of the *next* ASM-unit is completed, every created node is updated with feedback regarding the controller's progression through the higher order network:

6. A boolean *A* indicating whether the behavior associated with the node is reinforced (*A* = 1) or is inhibited (*A* = 0). (We will discuss this aspect of the model, which concerns its adaptive properties, in Section 2.4.).

A completed node may thus be defined as the tuple:


(4) $$\begin{array}{l} N=\langle P,V,\Delta m,C,Z,A\rangle \end{array}$$

The number of nodes in each ASM-unit begins at zero and grows to a maximum of *N*_*max*_ in a developed robot. After this maximum is reached, old nodes are destroyed to make room for new nodes. All of these nodes' data are used in future activations of the ASM-unit to contribute to future output of the mapping function. However, for now we will ignore the adaptive mechanism of the model and disregard the influence of the *C*, *Z*, and *A* components of the nodes, which are involved in that mechanism. We will return to this aspect of the model in Section 2.4.

#### 2.2.2. Motor Command Generation

At the same time as the generation of a new node to store state data, the model also generates a change-in-motor-state vector Δm which influences the current motor activity of the robot and is associated with the new node. This is done by finding a *parent node*, which is the historical node which represents a state most relevant to the current sensorimotor state of the robot. The parent node is found by a similarity metric which is applied to all historical nodes within a fixed distance of the current sensorimotor state in sensorimotor space, and the node which yields the greatest similarity value is classed as the current parent node. The behavior associated with the new node will be similar to the behavior associated with the parent node, and to reflect this the two nodes are regarded as having the same *class*. This is represented in-model with the new node's *C* component set to the same value as the parent's.

**Figure 7:**
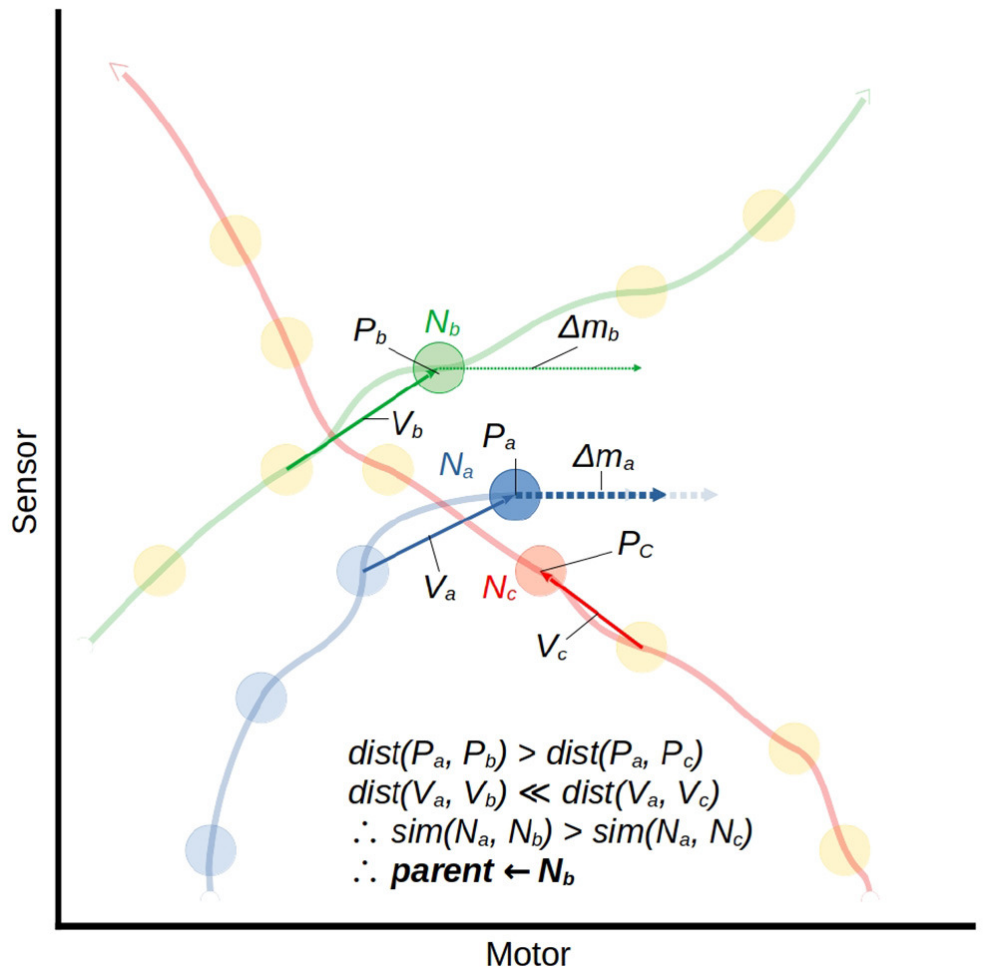
A visualization of the mapping function using the similarity metric described in Equation (5). This illustrates the moment in which node *N*_*a*_ is being created. The position in sm-space *P*_*a*_ and displacement from the previous node *V*_*a*_ will be compared to those of every nearby node. We isolate two of the three nearest nodes, *N*_*b*_ and *N*_*c*_, to compare. *N*_*c*_ is closest in space to *N*_*a*_, but the velocity of the trajectory associated with *N*_*c*_ is very different from that of the current trajectory. *N*_*b*_ is slightly further away, but the velocity of its associated trajectory is much more similar, so *N*_*b*_ is selected as the parent node of *N*_*a*_. Δ*m*_*a*_ is taken as the average between Δ*m*_*b*_ and the hypothetical vector which would put *N*_*a*_'s successor at the same motor state as *N*_*b*_'s successor. This is indicated by the pale arrows behind the Δ*m*_*a*_ arrow.

The similarity metric which finds the parent node is illustrated by example in Figure 7. Let us consider a node which has just been created *N*_*a*_ = 〈*P*_*a*_, *V*_*a*_, Δ*m*_*a*_, *Z*_*a*_, *A*_*a*_〉 and an arbitrary historical node *N*_*b*_ = 〈*P*_*b*_, *V*_*b*_, Δ*m*_*b*_, *Z*_*b*_, *A*_*b*_〉 We measure the similarity of the historical node to the current node as the weighted product of the Manhattan distance between their positions in sensorimotor space and the distance between their incoming vectors.


(5) $$\begin{array}{l} sim\left(N_{a},N_{b}\right)=-1\left(\overset{n}{\underset{i=1}{\sum }}|P_{a_{i}}-P_{b_{i}}|{\left(\overset{n}{\underset{i=1}{\sum }}|V_{a_{i}}-V_{b_{i}}|\right)}^{\omega }\right) \end{array}$$

Where ω is a fixed parametric weight which scales the relative importance of *V* compared to *P*.

Once a parent node has been identified, it is used to determine a change in motor state for the robot. The method for this is illustrated in Figure 7. The Δ*m* value for the new node is generated by taking a modulated form of the parent's Δ*m* value:


(6) $$\begin{array}{l} \Delta m_{a}=\frac{motor\left(P_{a}\right)-motor\left(P_{b}\right)}{2}+\Delta m_{b} \end{array}$$

Where *motor*(*P*_*a*_) refers to taking only the motor components of the sensorimotor position. In effect this produces an interpolation of Δ*m*_*b*_ and the hypothetical vector which would yield *N*_*a*_'s successor having the same motor state as *N*_*b*_'s successor. If a parent node cannot be found, either because this is the first activation of this ASM-unit, or because no historical states were sufficiently close to the current state, then Δ*m* is generated randomly, with each component of the vector selected from a normal distribution (μ = 0, σ = 0.03).

Once it has been generated, Δ*m* is used to determine a rate of change in motor state for robot over the next interval of τ:


(7) $$\begin{array}{l} ṁ=\frac{\Delta m}{\tau } \end{array}$$

In other words the robot's motor state changes linearly from time *t* to time *t*+τ so that the motor state shifts from *m*_*t*_ to *m*_*t*_ + Δ*m* over that interval.

### 2.3. Network-Level Model Architecture

We have discussed the design of an ASM-unit in isolation, and will now move on to how multiple ASM-units are linked together as a network as illustrated in Figure 4. As already mentioned, each ASM-unit spends only a limited period of time in a state of activation, and this state of activation regularly transitions from unit to unit. Transitions occur when the state of the system meets particular conditions, which depend upon either the robot's sensorimotor state or the duration of an activation. With a finite number of ASM-units in the network, walks through the network ultimately become cyclical, and this leads to the repeated activation of individual ASM-units which enable the history-based mapping functions to develop as they are applied.

The network, taken as a whole, defines the robot's behavior at a higher order than the immediate motor activations generated by an individual ASM-unit's mapping function: The complete activation of a specific ASM-unit reflects a directed transformation from one sensorimotor state to another (i.e., from one transition condition to another) over a discrete period of time, abstracted from the sensorimotor dynamics involved in producing that transition. In other words we may think of a complete activation of an ASM-unit as reflecting a performance of a discrete act (i.e., pushing a ball downwards), whereas the internal processes of each ASM-unit are reflective of the continuous sensorimotor dynamics that constitute that act (i.e., applying a certain amount of tension into the muscles as the surface of the skin feels a certain amount of pressure). Thus, similarly to the way that a set of sensorimotor trajectories captured in an individual ASM-unit reflect a set of regularities in a particular context of the agent-environment coupling, a repeated walk through the ASM-network reflects another set of historically-established regularities at a more coarse time scale. Having a multitude of ASM-units in the ASM-network produces a level of context-dependant and time-extended variability to the model's behavior: For any given sensorimotor state, one ASM-unit's mapping will likely give an output unique from any other ASM-unit. This means that the structure of the ASM-network, and the sequential order in which ASM-units are activated, is as fundamental to the behavior of the model as each independent mapping function.

**Figure 8:**
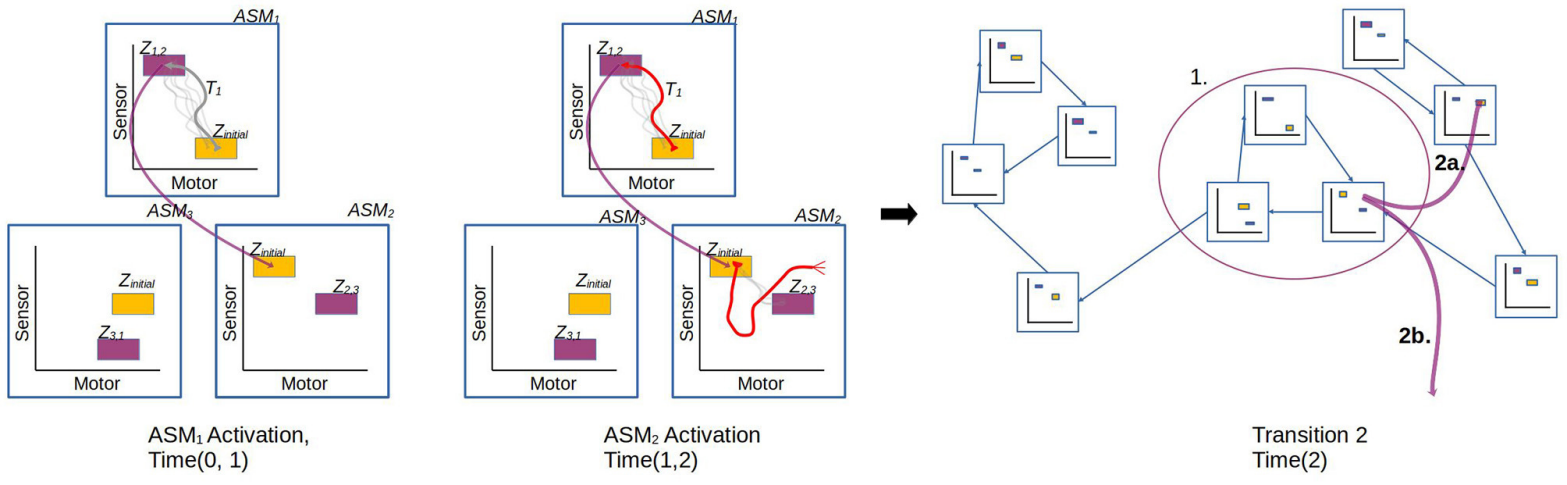
Illustration of the transition process involved when an active ASM-unit succeeds (*ASM*_1_) or fails (*ASM*_2_) to establish the necessary sensorimotor conditions to make a transition that is associated with stable behavior. After *ASM*_1_'s activation, the network transitions from *ASM*_1_ to *ASM*_2_, which have an historically established link. After *ASM*_2_'s activation, however, the conditions to transition from *ASM*_2_ to *ASM*_3_ are not present. The controller explores SM-space until a stable behavior is re-established. It could do this by either by transitioning to an ASM-unit which was not previously linked (2a.), or through random motor activity (2b.).

In the general case, the ASM-network topology is dynamic and may generate new ASM-units over time and establish new links between ASM-units, however the details of this are not relevant to the investigation presented in Section 3 which uses a static network, and thus we will save that description for future work. Here we will focus on how groups of ASM-units are linked as a network and how transitions occur from one unit to another. Figure 8 illustrates the different transition processes in a network. Each ASM-unit has a set of transition conditions *Z*, with each condition being associated with exactly one other ASM-unit. Each transition condition is defined as a hyperrectangular region in sensorimotor space with fixed upper and lower bounds along each dimension. Any time that the robot's sensorimotor state is inside one of these regions of the active ASM-unit, the transition condition is considered to be satisfied. When the condition is satisfied, activation of the current ASM-unit ceases and the ASM-unit associated with the transition condition becomes active. All ASM-units also define a set of initial conditions, which is simply the union of all of the transition conditions associated with that ASM-unit in *other* ASM-units in the network.

Transitions may also occur if the activation of a single ASM-unit has lasted for an over-extended period of time. The motivation for this is clarified in the next section based on the *principle of regularity* underlying the model's design. The limited time window is handled through a pair of parameters *t*_*g*_, and *t*_*h*_. The first defines a grace period, *t*_*g*_ = 8, in which only the active ASM-unit's transition conditions are checked. The second parameter defines a hard limit for the activation window, *t*_*h*_ = 16. In the times between *t*_*g*_ and *t*_*h*_ of an ASM-unit's activation, all of the network's initial conditions are checked as though they were transition conditions for the current ASM-unit. Finally, at time *t*_*h*_, activation of the ASM-unit is terminated immediately and the controller generates random motor activity until any ASM-unit's initial condition is satisfied.

When a transition occurs, the formerly active ASM-unit's recently created nodes are updated with information about the transition condition, as explained in the earlier description of nodes. This aspect of the model is motivated by the need to adapt to irregularities in the agent-environment coupling, and other principles of structural self-individuation. We will now discuss the former concern in detail, but hold back discussion on the latter for a future work.

### 2.4. Adaptive Mechanisms of the Model

At last we turn to the adaptive mechanism of the ASM-network. This mechanism produces a simple intrinsic goal for every activation of an ASM-unit, toward which it is biased to develop: To establish both sensorimotor and environmental conditions that are sufficient to allow the next ASM-unit to do the same for its own successor, thereby maintaining the established structure of the ASM-network as a whole. To explain this, we begin by temporarily stepping back from the technical description to discuss how behavior can be understood as adaptive and maladaptive in the context of the model.

**Figure 9:**
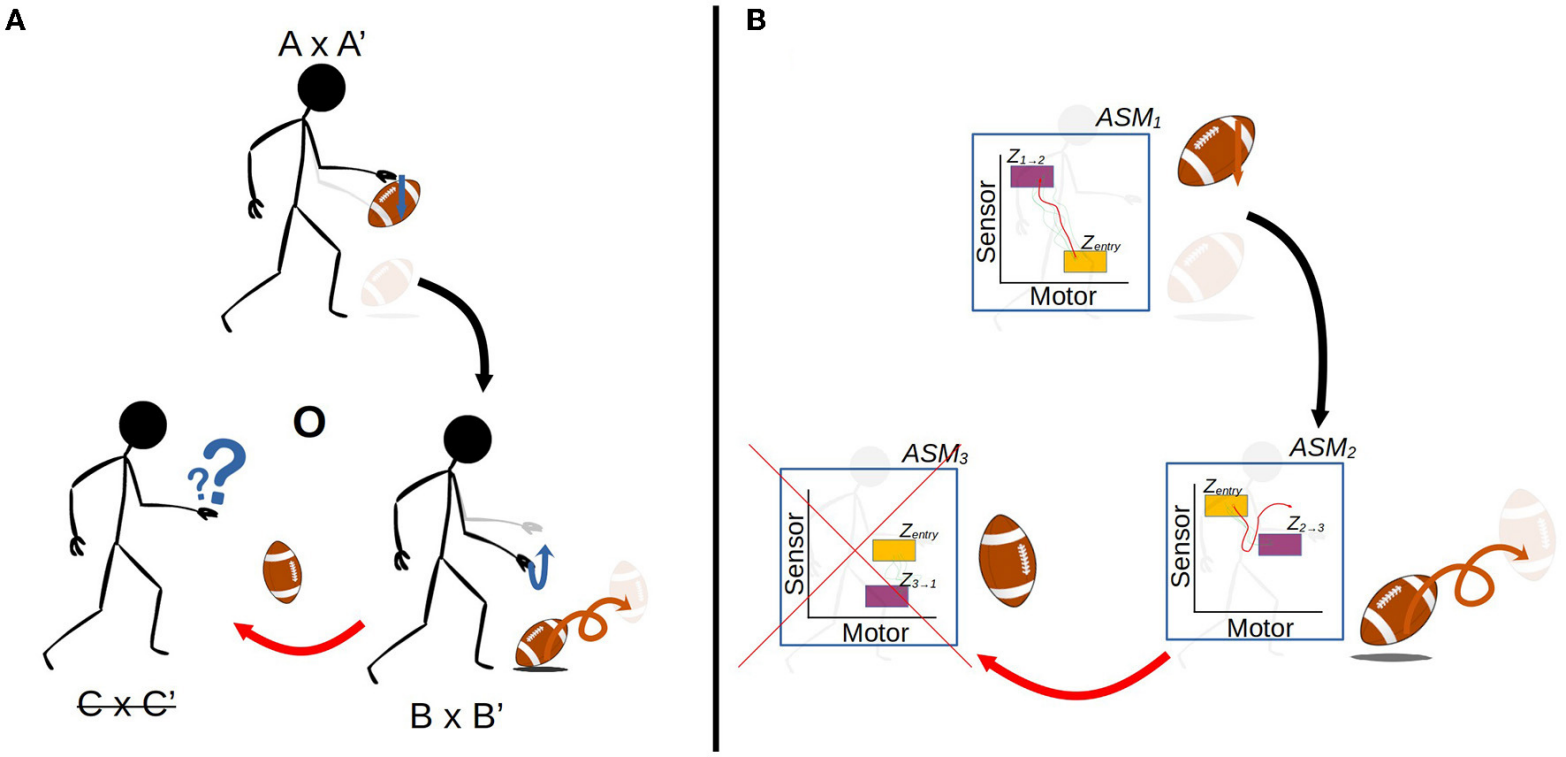
**(A)** A visualization of a disruption to the basketball-bouncing scheme. The agent-side dynamics remain the same, but the environmental support for the scheme is insufficient, specifically in terms of the shape and other physical properties of the ball which cause it to bounce differently off the ground. This disruption prevents the transition from B × B' to C × C'. **(B)** An idealized illustration of an ASM-network controlling a robot encountering that disruption. Although the sensorimotor regularities involved in pushing the ball downwards are compatible which the established scheme, as the ball bounces wildly the sensorimotor relationship becomes irregular, perhaps due to the variation in the robot's visual sensors. This prevents *ASM*_3_ from being activated as the robot is not in a suitable sensorimotor state.

Recall Figure 1, which presented an illustration of sensorimotor scheme associated with bouncing a basketball. In that scheme, there is an established structure of regularities in the agent's movements and perceptions, and in the way that the ball responds to and enables them. However, if a disruption is introduced to the scheme, say the ball is the wrong shape to bounce in the same way as a basketball, performance of the scheme will quickly go away. Figure 9A illustrates such a disruption to the environmental response structure. In that example, the coordination in which the agent prepares to receive the returning ball is disrupted when the ball bounces away in a way that a basketball would not have. The previously established regularities in the relationship between motor action and sensory stimulation do not hold. The same actions associated with receiving the ball are met with irregular sensations, perhaps an emptiness of the hand and a sight of the ball moving away. The enabling conditions for the next coordination are not met, and the agent is at a loss. Successfully adapting to this disruption would entail altering the dynamics of the interaction with the ball such that the various normative conditions that motivate the agent to bounce the ball remain satisfied. At the sensorimotor level, this would mean enabling the continued performance of subsequent and concurrent sensorimotor coordinations as they have been established through experience.

In order to adapt to a disruption such as the misshapen ball, the agent could either adjust its movements so that it can bounce the different ball in such a way that it returns to hand, or it could do something other than bounce the ball if it is misshapen—perhaps kick it instead. In the former case, adaptation occurs within the context of a sensorimotor coordination—the dynamics involved in transforming the state of the coupling from one set of enabling conditions to another may alter while the same organization of coordinations is retained. In other words the agent may attempt to reconcile the disruption with the pursuit of the same goal. In the latter case, adaption occurs at the schematic level, through new coordination structures providing compensatory progressions through the same scheme, or with the emergence of a diverging sensorimotor scheme with a different normative orientation. These processes could also occur in tandem to a greater or lesser degree.

Figure 9B illustrates an idealization of the scenario of a disruption playing out in the case of a robot controlled by an ASM-network, comparable to Figure 5 which illustrated the robot enacting the scheme without disruption. Just as the agent-environment coupling is not in a suitable state for enacting the next coordination, so is the ASM-network not meeting the conditions to allow the next ASM-unit to become active. Processes which compensate for such a disruption in ASM-network model could occur at both the unit and network level: Adaptation at the level of the coordination structure can be influenced through the reinforcement and inhibition of particular historical trajectories depending on how they relate to the resulting progression through the network. This alters the mapping functions and therefore the low-order dynamics of the coupling, while retaining the same higher-order sensorimotor transformations across sequences of ASM-unit activations. At the schematic level, adaptive processes can be influenced through the creation of new ASM-units in the network and new links between existing ASM-units. This allows new mappings to be generated and new transitions to occur, to accommodate new modes of agent-environment engagement. In this article, we focus purely on how the model's dynamics at the unit-level can adapt to maintain a pre-existing structure. The latter part of the adaptive process—how the structure of the network can generate dynamically—is equally important. However, we save that description for a future work as it is not a part of the investigation presented in Section 3.

The ASM-unit's adaptive mechanism is based on a *principle of regularity*. In a correctly functioning ASM-unit with an established set of historical trajectories, if the environmental state is sufficiently similar to its state during previous activations, then the sensorimotor trajectory produced by the ASM-unit's operation should also be similar to the trajectories produced by previous activations. By “similar trajectories” in this context we specifically mean two trajectories which begin within the same enabling conditions and reach the same set of transition conditions within a limited time window. This principle follows from the idea that because the controller is by design attracted toward repeating historical motor activity, the source of major deviations in a sensorimotor trajectory must be irregularities in the environmental response structure. Following from this principle, for a sequence of coordinations to be actively maintained over time, the stability of the environmental support structure must also be maintained. This provides a condition by which a sensorimotor trajectory may be evaluated in the context of enacting sensorimotor coordinations: Not only must there be regularity in the relationship between action and perception within a coordination, but that regularity must correlate with the stability of the environmental support for the next coordination. The model reinforces or inhibits trajectories based on whether that correlation appears to hold, based on the principle of regularity.

**Figure 10:**
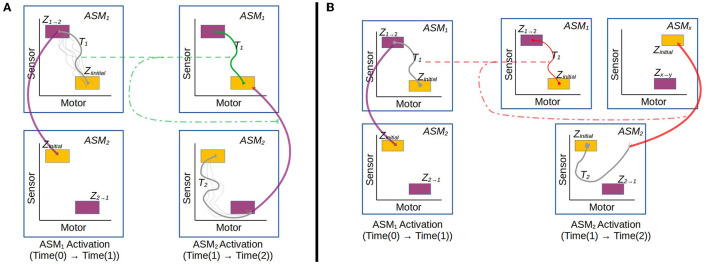
**(A)** Illustrates an instance of reinforcement. At the end of Activation 2 there is a successful transition from *ASM*_2_ to *ASM*_1_, and this leads to a reinforcement of the trajectory from Activation 1 which established the conditions for that successful transition. **(B)** By contrast illustrates an instance of inhibition. At the end of Activation 2 the established conditions to transition to *ASM*_1_ are not met, and activation passes to some other unit *ASM*_*x*_. This causes the trajectory from Activation 1 to be inhibited because it produced conditions which led to an unsuccessful transition.

Figure 10A illustrates the process of reinforcing an instance of a behavior. The model always reinforces any behavior which does not lead to a failure to produce a regular trajectory in the next ASM-unit. In other words if the sensorimotor trajectory over the course of an ASM-unit's activation is similar to historical trajectories, then we assume that the environmental state delivered by the *preceding* ASM-unit activation provided suitable support for the sensorimotor coordination. It follows that the activation of the preceding ASM-unit did not establish any instability in the environmental support structure, and therefore that trajectory should be reinforced to have an attractive influence on future behavior.

In a contrasting example, Figure 10B illustrates an instance of nodes in a trajectory being inhibited because they lead to a breakdown of the established sensorimotor regularities. It follows from a corollary of the principle of regularity that if the current sensorimotor trajectory is different from historical trajectories produced by the same ASM-unit, then sufficient environmental support was not established by the *preceding* ASM-unit (e.g., *ASM*_1_ in the figure) despite it achieving a suitable sensorimotor state. This means that the mapping associated with that ASM-unit, and the behavioral dynamics that it produces, are not sufficient to maintain the stability of the broader sensorimotor structure, because it produced regularities in the sensorimotor response that did not correlate with the stability of the environmental support structure. Therefore, the nodes associated with the dynamics of the preceding ASM-unit's last activation are inhibited, in order to alter the unstable dynamics. Additionally, the nodes in the current ASM-unit (e.g., *ASM*_2_ in the figure) which fails to transition to the expected next ASM-unit are also inhibited, as they too reflect dynamics which did not produce a stable engagement.

Let us return to the details of the implementation. Trajectories are reinforced or inhibited by setting the value of *A* component of every node involved in representing that trajectory. Recall that this component is simply a marker of this reinforcement property: if the trajectory is reinforced, then *A* = 1 for every node associated with that trajectory. If inhibited, then *A* = 0 for every node. Our explanation of the ASM-unit's mapping function in Section 2.2 assumed that all nodes were reinforced, but we now complete the explanation in the case where nodes may be either reinforced or inhibited. Recall that we previously stated that if *A* = 1 for all nodes, then the parent node is identified as the historical node which yields the highest similarity score in the metric given in Equation (5). When the adaptive component is included however, and *A* = 0 in some cases, the ASM-unit uses a filtering process to bias the system toward repeating behavior associated with the most relevant reinforced historical trajectory, even if there are several other more similar historical trajectories. The process may be best described algorithmically:

1. *sim*(*N*_*a*_, *N*_*i*_) (Equation 5) is applied to all nodes to find the node which produces the highest similarity score, call it *N*_*b*_.
2. If $N_{b}^{A}=1$, then *N*_*b*_ is regarded as *N*_*a*_'s parent node and the algorithm terminates.
3. Otherwise, a set **C** of node class labels is created such that $\text{C}=\left\{N_{b}^{C}\right\}$.
4. The node with the *next* highest similarity score is found, call it *N*_*c*_.
5. If $N_{c}^{A}=1$ and $N_{c}^{C}\notin \text{C}$ and $N_{c}^{Z}\neq N_{b}^{Z}$, then *N*_*c*_ is regarded as *N*_*a*_'s parent node and the algorithm terminates.
6. Otherwise, set $\text{C}=\text{C}\cup \left\{N_{c}^{C}\right\}$ and return to step 3 until the algorithm terminates or there are no more valid historical nodes for comparison.

Once the algorithm terminates, the Δm change-in-motor-state is generated as described earlier. This process causes the behavior of the robot to be directed toward repeating dynamics which ultimately supported successful transitions through the network, as well as actively avoiding those which failed. Essentially the addition of new, reinforced nodes representing an instance of a behavior increase the likelihood of that behavior's future performance by (1) increasing the diversity of states which attract the repetition of that behavior, and (2) by lasting longer than the nodes which came before them, given the finite capacity for nodes in an ASM-unit. By contrast, the addition of inhibited nodes reduces the likelihood of the same behavior being repeated in the future by negating the influence of reinforced nodes *via* the effect of the *C* and *Z* components.

This completes our description of the model as used in this investigation. The reinforcement and inhibition mechanisms produce a simple intrinsic goal for every activation of an ASM-unit, toward which it is biased to develop: To establish both sensorimotor and environmental conditions that are sufficient to allow the next ASM-unit to do the same for its own successor. We now present results of an investigation which demonstrate how this intrinsic goal can in turn produce more second-order goal-directed behavior in the robot which the ASM-network controls.

## 3. Investigation

We now demonstrate how an ASM-network can be used to control a robot which successfully learns to perform a task involving object discrimination. The parameters of the robot and environment are essentially equivalent to an experiment first presented by Beer (1996), in which agents were evolved to distinguish between circles and diamonds using the standard evolutionary robotics technique of evolving a continuous-time recurrent neural network controller using a genetic algorithm. The agents demonstrated their ability to distinguish between the shapes by colliding with circles while avoiding the diamonds. The task captures a fundamental capacity of any acting agent—in order to selectively interact with its environment, an agent must be capable of discriminating between different environmental features. We use this task as a first demonstration of the ASM-network's value in investigating goal-oriented adaptive behavior guided by the relationship between environmental and internal mechanisms, as opposed to extrinsic fitness functions.

In our version of the experiment no genetic algorithm or any other external optimization process is required. The properties of the environment are specifically arranged, and the ASM-network model is partially constrained, so that the stability conditions for the ASM-network's dynamics concurrently produce behavior which aligns with the ascribed norms of the task. Initially the robot's behavior is entirely random, but over time the robot develops an ability to scan the shape, identify the difference between circles and diamonds, and responds appropriately to the different shapes. Our results illustrate how our robots solve the task.

### 3.1. Experimental Setup

**Figure 11:**
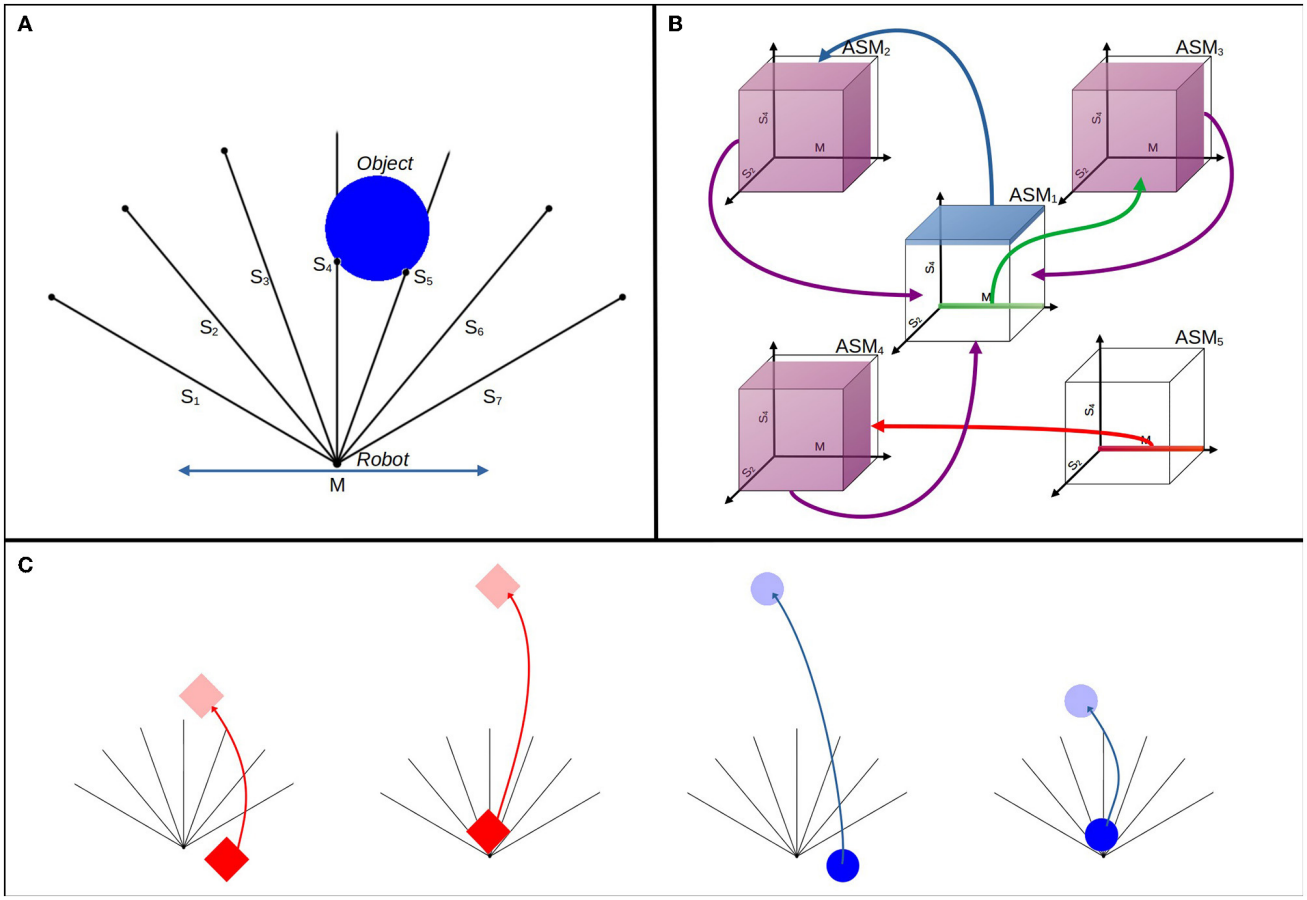
Experimental setup in three parts. **(A)** Illustrates the arrangement of the robot, its sensors, and an example of the circle descending. **(B)** Illustrates the topology of the network that controls the robot. Transition conditions are suggested in three dimensions, with arrows and color-coding indicating which ASM-unit is enabled by each transition condition. These conditions are discussed more precisely in the body text. **(C)** Illustrates the different ways that the object types react to hitting the robot or the arena floor.

Figure 11 illustrates the experimental setup. A robot with seven ray sensors and one bi-directional motor is situated in a 2 dimensional arena. The rays are spread evenly with an angle of $\frac{\pi }{9}$ radians between each, with three on either side of a central ray pointing directly upwards. The motor allows the robot to move horizontally with a velocity ranging between −30 and 30 units per second. The arena has a width of 300 units and a height of 300 units, and periodic boundaries. At the start of a simulation the robot begins at position (150, 0) in this arena. An object, which may initially be either a circle or a diamond shape is positioned in the arena. The object enters the arena at 100 units above the robot vertically and offset between −50 and 50 units horizontally from the robot. Circle objects have a diameter of 36 units, and diamonds have a side length of 36. The objects falls at a rate randomly selected between 12 and 16 units per second. The robot's sensors are stimulated whenever the ray intersects with the falling object, with the sensor activation modeled as a continuous scalar which linearly increases from 0 if the intersection point is at the tip of the ray, up to 1 if the intersection is at the position of the robot.

A single run of the experiment continues until there have been at least 2,000 descents of both circles and diamond objects. The results here are based on 64 runs. During a run, the object falls directly downwards, while the robot moves around the arena freely. When the object collides with the robot or the bottom of the arena, it returns back toward the top of the arena immediately, responding differently if it is a circle or a diamond. This is illustrated in Figure 11C. If a circle collides with the robot, it returns 100 units vertically, whereas if it collides with the bottom of the arena it returns 300 units. The inverse is true for the diamond, it returns 100 units when it hits the bottom of the arena and 300 units when it collides with the robot. When the object returns, its downward velocity randomly resets to a new value from the same possible range, the object's horizontal offset is randomly reset, and the shape has a chance of swapping to the other type with a probability of *p* = 0.5. This closely resembles a succession of resetting trials in an evolutionary robotics framework, but we emphasize that a single robot remains active over the course of the entire run, and the single ASM-network develops its history over the run. The continuity of a run is critical for two reasons: (1) The ASM-network needs to build a history of behavior over time for it to develop toward solving the task; (2) There is a consequential difference for the robot between colliding with an object and missing an object, in that the object returns to its sight more or less quickly depending on its shape and whether it collides or not (i.e., diamonds returning either 300 or 100 units up and the opposite property for the circles). The significance of these differences is discussed in detail in Section 4.

Each ASM-unit in the network has a sensorimotor space with eight sensorimotor dimensions (*S*_1..7_ ∈ [0, 1], *M* ∈ [0, 1]), coinciding with the seven sensors and one motor ($R^{S_{1..7}}\in \left[0,1\right]$, *R*^*M*^ ∈ [−30, 30]) of the robot, such that:


(8) $$\begin{array}{l} M=\frac{R^{M}+30}{60} \end{array}$$

(9) $$\begin{array}{l} S_{x}=R^{S_{x}} \end{array}$$

The network is constrained to support the fulfillment of the task. The network has a fixed arrangement of five units, with pre-given transition conditions and links between each. These transition conditions are associated with the potential sensorimotor states of particular stages of the desired functional behavior, e.g., when the robot sees any object, when the robot collides with any object. This scaffolds the development of functional behavior and constrains which habits are potentially viable, but it does not define the behavior of the robot, as all of the motor dynamics are produced by the ASM-unit mapping functions, which begin undefined as there is no history for them to respond to. The way in which these constraints scaffold specific functional behavior is explained in Section 4.

The topological arrangement of the network is illustrated in Figure 11B, but due to the dimensionality of the sensorimotor space the transition conditions are only able to be suggested in an image. We define them precisely here. *ASM*_1_ has 2 separate transition conditions linked to *ASM*_2_ and *ASM*_3_, respectively. The transition condition *Z*_1,2_ (i.e., condition for the transition from *ASM*_1_ to *ASM*_2_) is defined as follows:


(10) $$\begin{array}{l} Z_{1,2}:\left\{M\in \left[0,1\right],S_{1,2,3,5,6,7}\in \left[0,1\right],S_{4}\in \left[0.98,1\right]\right\} \end{array}$$

Where *M*∈[0, 1] means that the motor state as represented in the ASM-unit may be anywhere between 0 and 1 to satisfy the condition. *S*_*x*_ refers to the same for each sensor. Note in particular that *S*_4_ is different from the others. Practically, this means that the transition occurs whenever the robot's central sensor is very highly stimulated, and all other sensors and the motors may be in any state. This condition would occur whenever the object collides into the front of the robot. The other transition condition is:


(11) $$\begin{array}{l} Z_{1,3}:\left\{M\in \left[0,1\right],S_{1,2,3,4,5,6,7}=0\right\} \end{array}$$

Which means that this condition is satisfied if and only if every sensor is at 0, i.e., the robot cannot detect the object.

*ASM*_2_ has two transition conditions which are both linked back to *ASM*_1_:


(12) $$\begin{array}{l} \begin{array}{c} Z_{2,1}^{a}:\left\{M\in \left[0,1\right],S_{1,3,4,5,6,7}\in \left[0,1\right],S_{2}\in \left[0.01,1\right]\right\}\\ Z_{2,1}^{b}:\left\{M\in \left[0,1\right],S_{1,2,3,4,5,7}\in \left[0,1\right],S_{6}\in \left[0.01,1\right]\right\} \end{array} \end{array}$$

Which means that the conditions are satisfied if either the *S*_2_ or *S*_6_ sensors are at least slightly activated. In practice, at least one of these conditions is satisfied if the object is anywhere in the majority of the coverage of robot's sensory field, although not if for instance the object is moderately far to the left or right, or immediately in front of the robot at a long distance.

*ASM*_3_ has two transition conditions which are the same as those in *ASM*_2_, such that $Z_{3,1}^{a}\approx Z_{2,1}^{a}$ and $Z_{3,1}^{b}\approx Z_{2,1}^{b}$. We use the approximation to reflect that although the ranges are the same, the transition conditions are not identical because the sets require different ASM units to be active. *ASM*_4_'s transition conditions are also defined similarly to other units, such that $Z_{4,1}^{a}\approx Z_{2,1}^{a}$ and $Z_{4,1}^{b}\approx Z_{2,1}^{b}$, and finally *ASM*_5_'s transition condition is *Z*_5,4_ ≈ *Z*_1,3_. In Section 4, we discuss how this arrangement relates to the behavior that the controller produces in more detail.

### 3.2. Results

**Figure 12:**
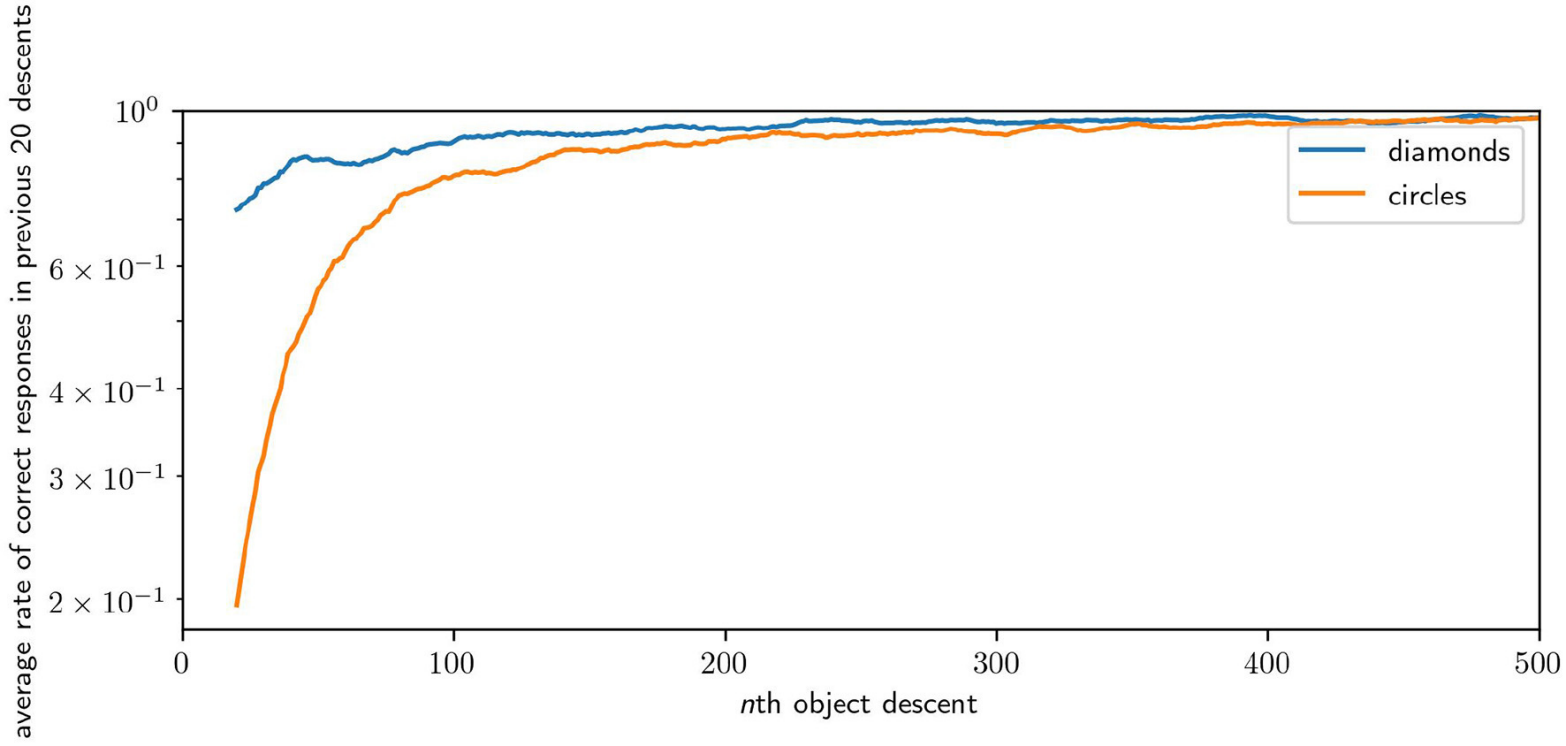
Plot of the improving performance of robots, averaged across 64 runs. Although this figure only shows the first 500 interactions with each shape because most of the development occurs early, performance continued to improve incrementally for the remaining 1,500 interactions.

We measure the performance of a robot in the task by looking at how many times the robot responded “correctly” in a window of the most recent descents of each shape. Figure 12 illustrates the average performance over time over 64 runs. The plot samples every 20th descent of either circles or diamonds, with each point giving $\underset{i}{\sum }\frac{c_{i}}{20\times 64}$, where *c* is the number of correct responses in the previous 20 descents of a shape for the *i*th robot. Across 64 runs, the average performance of the robots for the first 20 descents for circles is 0.37 (i.e., they catches circles 37% of the time) and for the first 20 descents of the diamond is 0.76 (i.e., they avoid diamonds 76% of the time). The average performance in the last 20 descents for circles is 0.97, and for the last 20 descents of diamonds the performance is 0.99. Performance improvement is rapid, reaching an average of over 0.9 for both shapes within 200 descents, and reaching peak performance after 1,000 descents. Performance for diamonds is higher, especially at the start, because of the greater likelihood of missing an object by chance compared to colliding with that object. Catching involves precise positioning, whereas avoiding can be accomplished in many equally good ways. These results illustrate that the robot is capable of learning how to effectively discriminate between diamonds and circles.

The robot is capable of reliably solving this specific task because the topology of the network is arranged such that the behavior involved in maintaining the established links in the network is necessarily also behavior which solves the task. In all robots we observed a direct correlation between the rate of successful transitions between ASM-units and the rate of the robot's correct responses to the object shapes. Crucially though, the pre-given topology and environmental conditions are insufficient to define for the robot the actual sensorimotor dynamics involved in solving the task, i.e., how to move around the environment in such a way that it can identify the different shapes and collide or avoid as appropriate. To learn these dynamics, the robot must engage with the environment over time, and over the course of this engagement the model's adaptive mechanisms reinforce those dynamics that support the transition conditions within the network and inhibit those that lead to violations of those conditions. The maps of each ASM-unit, most critically *ASM*_1_, develop in such a way that the robot's behavior consistently establishes the enabling conditions of each ASM-unit in a suitable sequential order. In *ASM*_1_ this means that the mapping must produce dynamics which differ when encountering differently shaped objects, as the environmental conditions have been set such that disruptions will occur elsewhere in the network if the robot interacts with the objects incorrectly. This means that the bulk of the learning process that occurs over the course of an experimental run is in the development of *ASM*_1_'s map.

**Figure 13:**
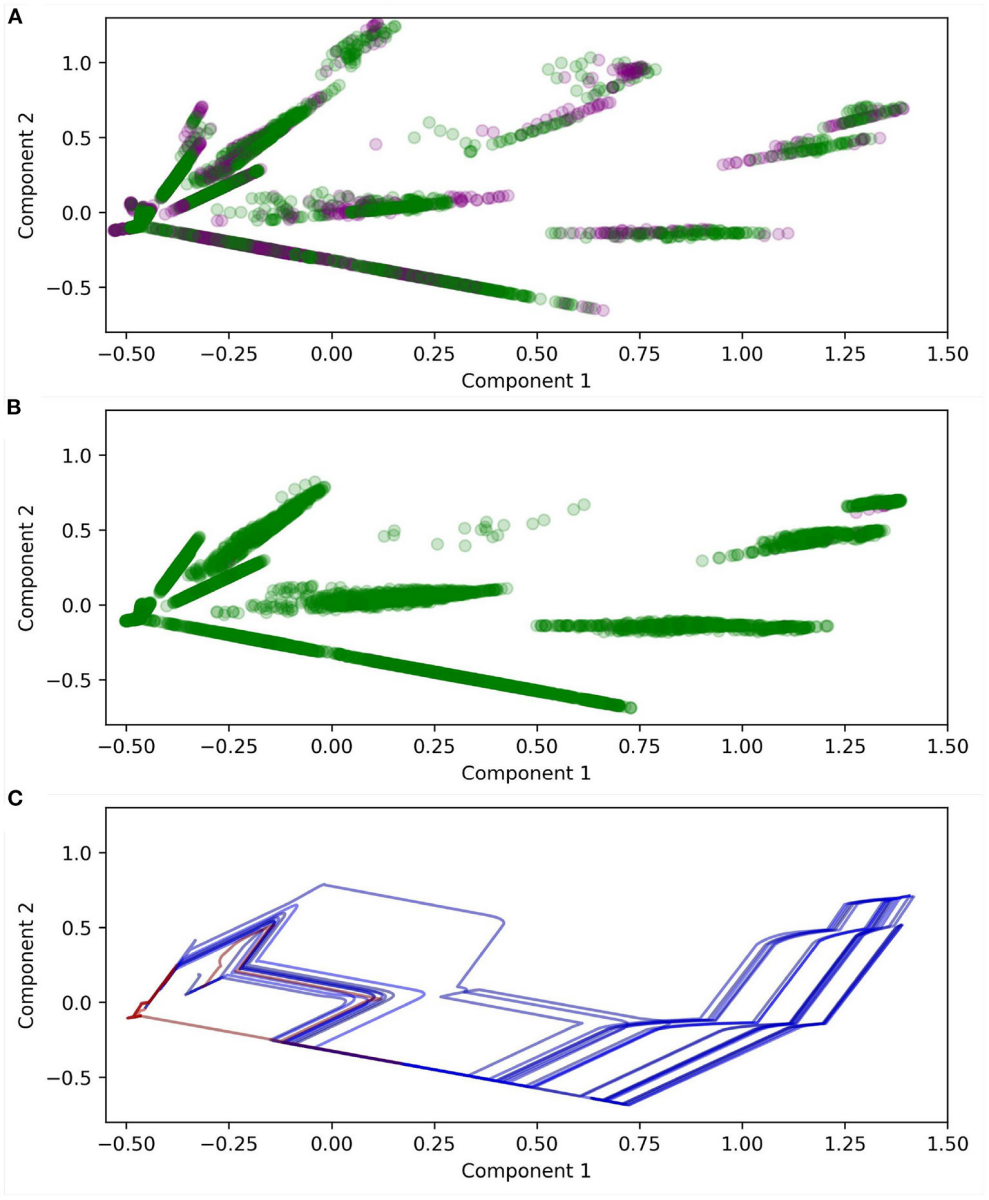
Projections of *ASM*_1_ derived using principle component analysis. **(A)** Illustrates the positions of the nodes after 100 descents of the object. Purple markers indicate inhibited nodes, green indicates reinforced nodes. **(B)** Illustrates the positions of nodes after 2,000 descents. **(C)** Illustrates the sensorimotor trajectories of the interactions with the last 10 descents of each shape. Blue indicates circles and red indicates diamonds. Note that the motor dimension is ignored because it confounds the interpretability of the plot. The rapid jumps in the plots are due to individual sensors suddenly becoming active or inactive as they intersect with the object.

Meaningfully visualizing the maps themselves, and how they change over time, is challenging due to their dimensionality. Figure 13 illustrates a projection of the states of *ASM*_1_ for one robot using principle component analysis. Note that Component 1 = [0, −0.03, −0.03, 0.04, 0.18, 0.44, 0.64, 0.59] and Component 2 = [0, −0.01, −0.01, 0.20, 0.66, 0.56, −0.32, −0.29]. The plot compares node positions in the early and late stages of the robot's development and highlights important differences. Firstly, almost all nodes are reinforced by the end of the robot's development. Secondly, there are subtle change in the distribution of the nodes over time, and by extension the mapping: One clear example is that inhibited nodes (purple) tend to be clumped together around (0.73, 0.9), in the early stages of development, and by the later stage of development most of the nodes in that area have disappeared. This suggests that the inhibited nodes successfully dissuade the continuation of trajectories which approach that region of sensorimotor space. Figure 13C illustrates the projected sensorimotor trajectories of the last 10 interactions with each shape for the same robot, indicating the way that the trajectories for different shapes diverge around (0, −0.25), and end in different regions of sensorimotor space.

**Figure 14:**
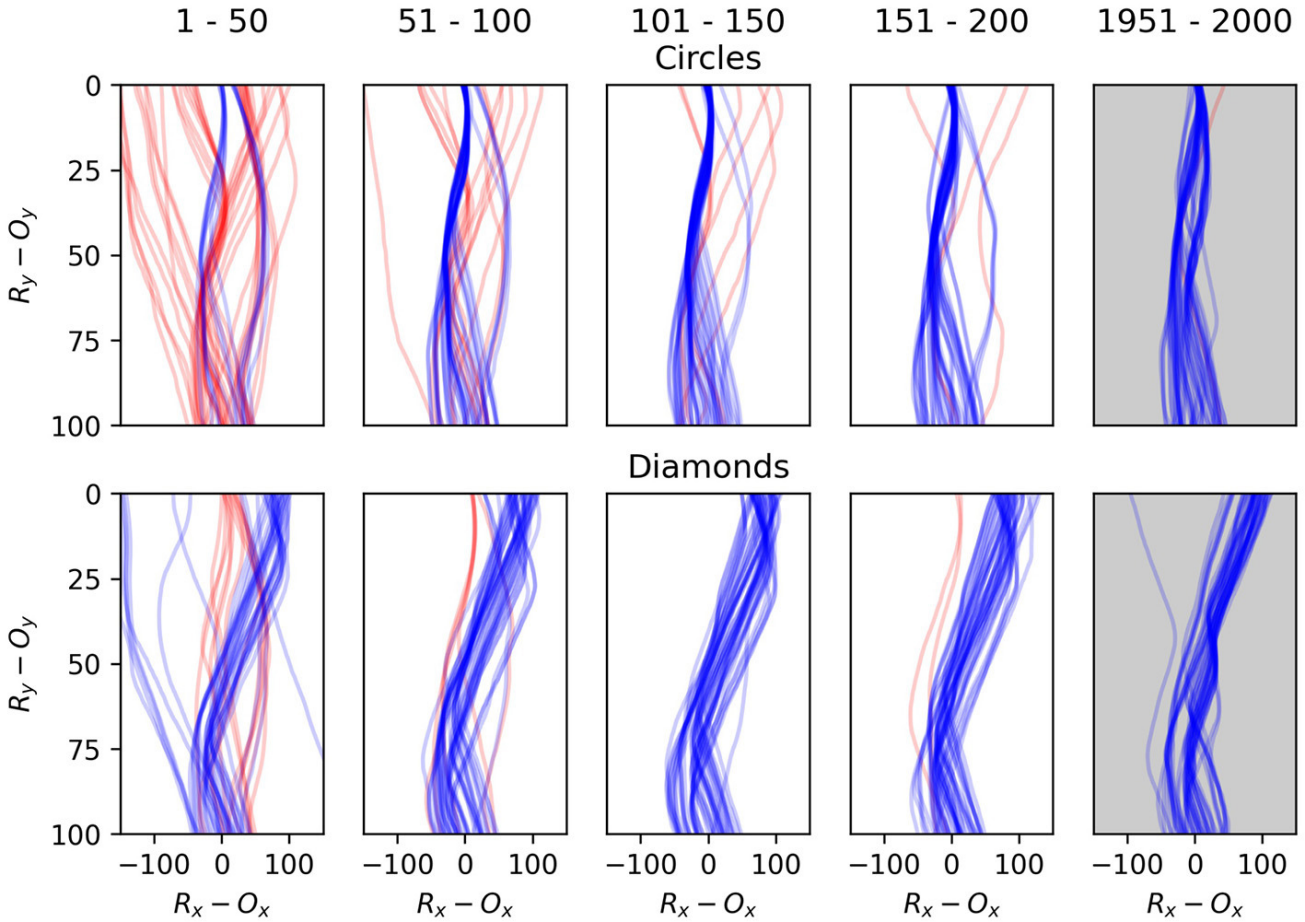
Plots illustrating the behavior one robot as it interacts with the falling objects. The first column shows the robot's first 50 interactions with circles in the top row and diamonds in the second row. The second column shows the second 50 interactions and so on. Each line illustrates a single interaction with an object from the moment that it is 100 units above the robot until the moment the object either collides with the robot or the bottom of the environment. Blue lines indicate that the object ultimately did the correct thing as per the task description, i.e., it collides with the circle or avoids the diamond. Following each plot from bottom to top shows the relative horizontal positions of the robot and object as the object descends toward the robot. The object's position is always at 0 on the x-axis, so a point at x = 100 indicates that the robot is 100 units to the right of the object, and gives no indication of the robot's absolute position in the environment.

**Figure 15:**
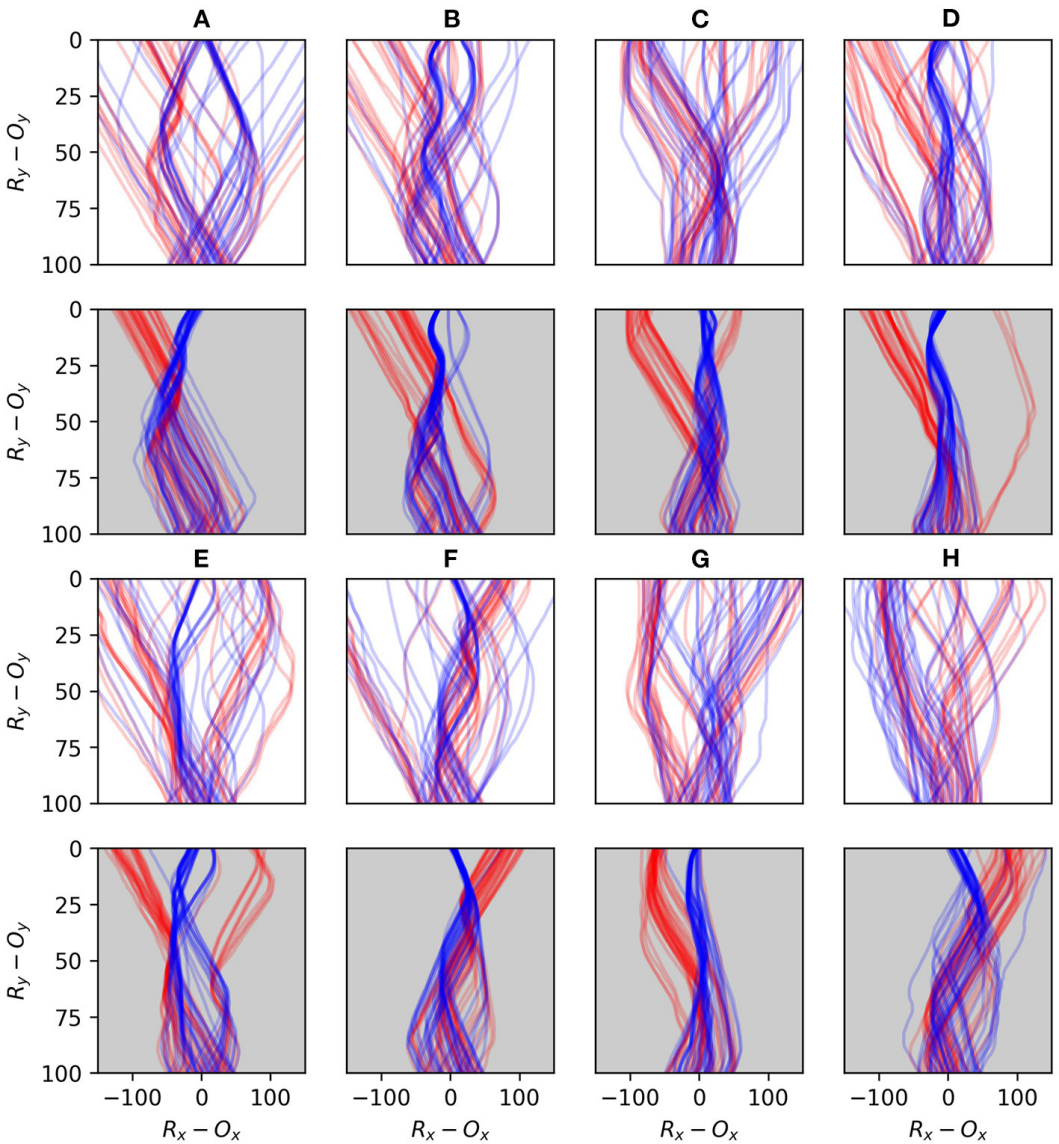
Plots comparing the early and late stages of development for eight randomly selected runs. The first and third rows illustrate the first 40 interactions with each shape type for 8 different robots **(A–H)**, and the second and fourth rows show the last 40 interactions for the same robots. Blue lines indicate interactions with circles and red lines interactions with diamonds.

The relative positions of the robot and object over time as the object descends through the robot's sensory field provide a better illustration of the robots' development. Figures 14, 15 illustrate examples of these trajectories. Figure 14 demonstrates the development of a single robot, which we selected to exemplify the way in which the adaptive mechanisms of the model contribute to particular dynamics becoming more stable than others depending on how well they support the network-level organization. The plots represent the robot's horizontal position relative to the object, from the moment the object is at a height of 100 units until the moment the object reaches height of 0. In each encounter the initial conditions of the object vary, in terms of speed and displacement, within the parameters already discussed. The top row illustrates the first 200 encounters with a circle, and then the last 50 encounters. The second row illustrates the same for encounters with the diamond. The majority of the improvement occurs rapidly, in the first 50 encounters. We can also see that more subtle developments continue, most notably a kind of behavior which leads to occasional narrow misses of the circle becomes less frequent between the 50th to 200th encounter. The final trajectories shows how the developmental process has continued over the longer term to exaggerate the differences between responses to the different object shapes, and to increase the consistency of responses to a particular shape, especially in the case of diamond encounters.

Figure 15 demonstrates the performance of eight randomly selected robots, contrasting the early stages of their interactions with the objects against the late stages. Every robot displays a tendency to transition from an initially sprawling set of different trajectories to a significantly more concentrated set of trajectories in the later stages. Given that the object appears at a random position with respect to the robot, a typical strategy emerges which involves the robot moving to approximately the same position relative to the object in each encounter, before the responses to the different shapes diverge. All of the robots display some variation of a behavior involving sweeping multiple sensors across the object in either direction before the responses diverge. Beer observed that similar foveate-scan-decide strategies were typical in his evolved CTRNN-controlled robots. This suggests that these kinds of responses are particularly attractive for this task even when the adaptive mechanisms producing such behavior are distinct.

### 3.3. Auxiliary Results

**Figure 16:**
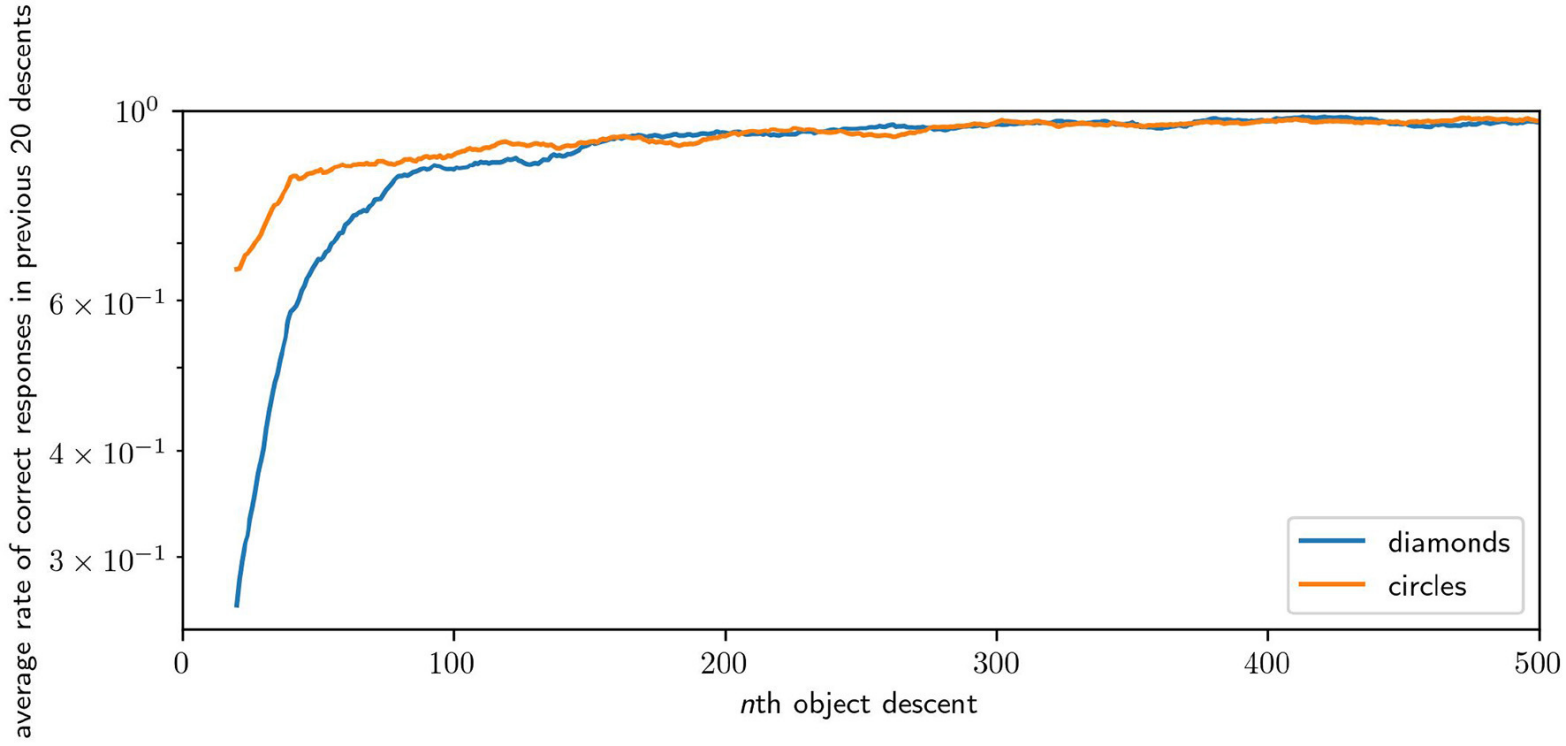
Results of an inversion of the default task, in which the responses properties of diamonds and circles are swapped. This causes the robots to develop to avoid circles instead of diamonds and *vice versa*. Results are presented as per Figure 12, note that the learning rates are equivalent but the robots take longer to respond correctly to diamonds instead of circles in this case.

In an auxiliary experiment we, performed 16 runs in which robots were only ever exposed to diamond objects. There we observed that a dominant behavior is for the robot to immediately and continually moving at full speed in one direction or the other, thereby missing the diamond by some distance. This is a very simple behavior for the robot to discover by accident as it simply involves keeping its motor state around its maximum or minimum regardless of sensory state. This behavior also appears in the early stage of Figures 15A,D–F, but is lost by the later stages. This suggests that such a behavior is less stable when circles are also present, as it limits the robot's ability to identify the shape type and respond appropriately. This provides an example of the space of viable habits being constrained by the contrasting properties of the shapes. Finally, we performed 64 runs in which the responsive properties of diamonds and circles was inverted. The average successful performance rate over the course of all robots' development is presented in Figure 16.

## 4. Discussion

### 4.1. How Can the Robots Perform the Task?

Our results present a model, based on enactive principles of sensorimotor contingency theory and habit, which allows a robot to learn to perform a specific cognitive task of object discrimination without a functionally-oriented reward mechanism. The model as presented in Section 2 is a generic medium which specifies a whole suite of dynamics with certain kinds of attractors. In the experiment presented in Section 3, we apply some constraints to that medium which establish the viability conditions of a particular sensorimotor organization, such that the internal norms of the system align with the ascribed norms of task, that is to avoid diamonds and collide with circles. This allows a generic adaptive mechanism, directed toward satisfying those internal norms, to also shape the behavior of the robot to satisfy the requirements of the task. In natural systems, a web of evolutionary and developmental processes all serve to shape the sensorimotor organization of an agent in a manner that produces an alignment such as this, while we have engineered the alignment with a specific set of constraints utilizing our knowledge of the task and system. How exactly does the process of maintaining this particular sensorimotor structure align with adaptively regulating behavior in terms of a functional task?

**Figure 17:**
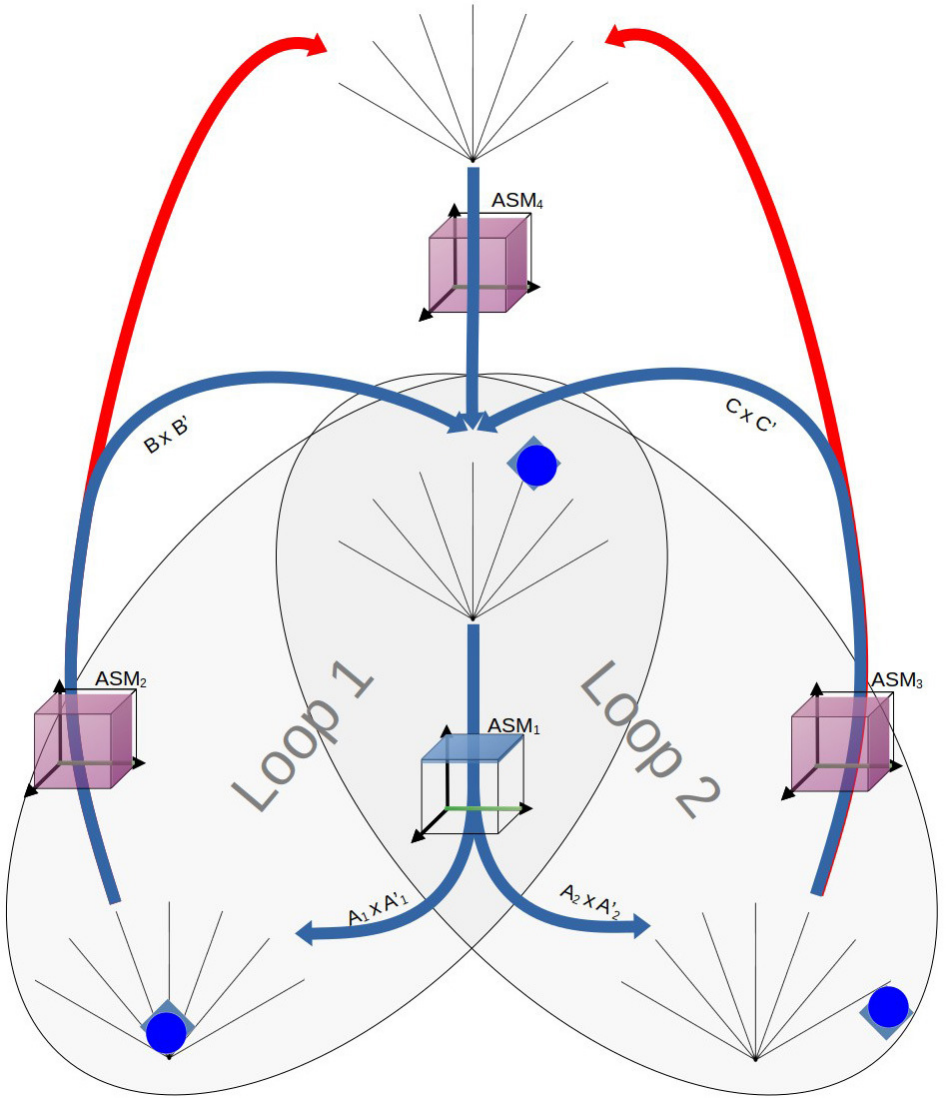
An illustration of the robot's arrangement of sensorimotor coordinations as they relate to the ASM-network. Successful performance of the task aligns with the robot proceeding through performance of Loops 1 and 2 without disruption. Over time *ASM*_1_ develops such that only Transition_3_ occurs when diamonds are present and only Transition_2_ occurs when circles are present. Some simple adaptive processes also need to take place in *ASM*_2_ and *ASM*_3_ such that they consistently lead to the robot re-discovering the object as it returns into view. Note that *ASM*_5_ does not feature as its only purpose in the network is to provide suitable initial conditions for *ASM*_4_.

Figure 17 illustrates the relationship between the enabling conditions of an organization of sensorimotor coordinations as they relate to the structure of the ASM-network used in the experiment. We conceptualize an agent performing this task as alternately enacting a pair of habitual behaviors, which, following (Egbert and Barandiaran, 2014), we understand as simple loops of sensorimotor coordinations:

1. In Loop 1 the interaction progresses from the robot detecting the object at long range until it collides with the object (A_1_ × A_1_'), and then from there until the robot detects the object at long range once more (B × B').
2. In Loop 2 the interaction progresses from detecting the object at long range until the object leaves the robot's sensor range (A_2_ × A_2_'), and then until the robot detects the object at long range once more (C × C')

We label A_1_ and A_2_ as such because they share an enabling condition but involve diverging sensorimotor trajectories to reach different transition conditions. Thus, we have two partially overlapping loops of sensorimotor coordinations A_1_ × A_1_' → B × B' → A_1_ × A_1_' and A_2_ × A_2_' → C × C' → A_2_ × A_2_. As we have discussed in Section 1, we may think of the continuing sequential satisfaction of the enabling conditions in these loops as the conditions of viability of a sensorimotor habit. By design, our ASM-network medium is oriented toward adapting behavior to maintain such viability conditions. Transition conditions between *ASM*_1_, *ASM*_2_, and *ASM*_3_ are associated with sensorimotor states associated with detecting objects, colliding with objects, and losing detection of objects, while *ASM*_4_ is associated with conditions that only occur when the expected progress through the loops is disrupted. Thus the pre-given structure of the ASM-network in Figure 11 imposes this kind of arrangement of sensorimotor coordinations onto the robots, and as such the sequential fulfillment of the coordinations' enabling conditions as its task. However, those habitual viability conditions are completely agnostic to the different sensorimotor properties of interacting with the shapes, and thus do not sufficiently explain the robots' functional fulfillment of the object discrimination task. It is the different properties of diamonds and circles, with respect to what happens when they collide with either the robot or the bottom of the arena, that imbues the shapes with intrinsic relevance with respect to these goals. Specifically, colliding with a diamond causes a delay in returning the object which disrupts the progress of Loop 1, and likewise missing a circle leads to a disruption Loop 2. Since the ASM-network's adaptive mechanism is geared toward avoiding behavior which produces disruptions, i.e., behaviors which are non-viable with respect to maintaining the arrangement of coordinations, the difference between the objects will drive the robot to respond differently to the two shapes. An interesting consequence of this is that if we invert the properties of circles and diamonds in terms of how they respond to collisions and misses, then robots with the same ASM-network parameters instead learn to seek diamonds and avoid circles. That the functional behavior produced is a equally a consequence of both the internal dynamics of the agent and dynamic properties of the environment highlight the value of this kind of experimental approach.

### 4.2. What Do the Robots Learn Autonomously?

We have discussed how the particular network used in the experiment produces an alignment between the internal mechanisms of our robot and the ascribed norms of the task. But since we achieve this alignment through directly engineering a set of constraints, what exactly is left to the robot to learn autonomously? In functional terms, the robot has only been given a structure of sensorimotor conditions that it needs to repeatedly satisfy in order to maintain stable behavior. It must learn that there is a difference in the way that the two shapes impact that stability, that the difference corresponds with particular perceptual characteristics of interacting with the shapes as they descend, and how to act in response to those different characteristics so that it avoids interactions which destabilize its sensorimotor structure. While the arrangement in Figure 17 are a consequence of the network structure illustrated in Figure 11B, the development of suitable ASM-unit mappings which satisfy this arrangement is comparable to the optimization of weightings in terms of task fitness in an Evolutionary Robotics approach.

We can explore this further to clarify our model's relationship to the theoretical concepts of sensorimotor contingencies and habit mentioned in Section 1. Our constraints on the network establish the parameters of the relationships between a set of sensorimotor coordinations that are necessary for those coordinations to be stable, but it does not establish the actual sensorimotor dynamics that constitute those coordinations. While the general effect of action on perception is implicitly established in the characteristics of the robot and shapes, closing the causal loop to establish the effect of perception on action can only be established through interaction between robot and environment. The development of those agent-side dynamics will vary based on whatever specific environment-side dynamics it encounters. The environmental dynamics can vary in terms of four properties, all of which change the perceptual character of interacting with the objects from the agent's perspective. These properties are: (1) the different shapes of the objects; (2) The different mechanical properties of the objects (i.e., what happens upon collision); (3) The different speeds of the objects; (4) The different initial displacements of the objects. With our privileged view of the system we know that the differences in properties 1 and 2 are meaningful with respect to the norms of the system, while 3 and 4 are not, but this is not made explicit in the experimental setup, i.e., the adaptive mechanism is not tuned to respond to those properties in the same way that a fitness function defines the relevant properties of the world. Through interacting with the environment over time, these relevancies nevertheless become expressed through the robot's behavior.

In developing stable habitual behavior, the robot effectively learns that it needs to respond to the different shapes differently and how to make those different responses. While it is not responding like this, the internal dynamics of the controller will be in flux since the agent-side dynamics of the coupling become altered when particular trajectories are inhibited. Stable habitual behavior entails the performance of a habit continually re-establishing the conditions of its own re-performance, but due to the instability of the controller the conditions for a particular way of performing a habit may be lost over time even if the same initial sensorimotor state is established. The results illustrate that the robots generally developing the foundation of a stable behavior within a few dozen interactions, but beyond this the behavior is refined over time as the robot generalizes that distinction between properties 1 and 2 across the variations produced by properties 3 and 4. This refinement coincides with a gradual improvement in task performance after the first, relatively rapid phase of acquiring a generally successful strategy. The behavioral refinement over time reflects an individuation process in two separate habits (i.e., robot-diamond interactions and robot-circle interactions) becoming more distinct from one another to avoid interference between the two, e.g., suddenly switching to an established seeking behavior while in the process of avoiding because the dynamics of each resonate too similarly with a particular context. Although this is only a limited form of individuation—the distinction between the structures of the habits is already present, only their constitutive dynamics become more distinct—it nevertheless points to interesting developmental processes which occur even within this constrained model.

### 4.3. Limitations and Future Work

A criticism may be made of our investigation that the constraints and carefully arranged properties of the experiment mean that the model's internal adaptive mechanism serves an analogous function to an external optimization process such as an evolutionary algorithm. While this is the case here, because we are imposing a specific behavior on the system, the crucial difference is that our model would still have an adaptive and developmental gradient in the absence of such constructions. In the typical evolutionary approach a specific functional behavior is attractive in its own terms, *via* the fitness metric. However in our approach the particular functional behavior is made attractive through the relationship between the environmental dynamics and the internal processes of the robot and controller. Attractive behaviors will still arise for any specification of environmental dynamics and be meaningful in these terms.

Nevertheless, it is worth discussing the consequences of the network constraints. In particular, the robot's capacity to solve the task as we expect it to do so relies in part on the fact that it is incapable of assimilating the “wrong” environmental support into its sensorimotor structure. In other words, the dynamics associated with the diamond disappearing from the robot's view for much longer when it collides with the robot are always treated as disruptive regardless of context and of how many times it occurs, and *vice versa* for the circle. This high-level rigidity limits the ways in which the robot may adapt. In discussing the development of sensorimotor schemes in human development, Piaget discussed three different classes of adaptive processes of how instances of this disruption are resolved over time (Chapman, 1992; Boom, 2010): (1) the disruption is ignored without altering behavior; (2) The behavior alters to compensate disruptions which have previously been encountered, or (3) potential disruptions are anticipated and behavior is altered so that the disruption is not encountered at all. The second process would most accurately describe that which is occurring in the robots in this investigation, while the others are not possible within the constraints that we have placed. The combination of these kinds of adaptive process is a key part of open-ended, autonomous development that is neicessary for sensorimotor agency. The obvious next step in terms of using the ASM-network to investigate sensorimotor agency is to remove the constraints at the network level, in a manner that allows precarious, self-maintaining structures to develop dynamically at that level.

Although the constraints we have placed on the model in this investigation limit the kinds of habits that may form autonomously, they allow for an analytically tractable investigation to demonstrate some of the model's capabilities. Our results provide a demonstration that the ASM-units are effective in producing behavior which supports the maintenance of a networked arrangement of such units that reflects a structure of sensorimotor coordinations. Furthermore, this alone is sufficient to produce a form of minimal cognitive behavior. However the model is also sufficiently generic that it is not necessary to have a pre-given network arrangement, engineered to align with a specific function, in order to produce coherent behavior. This opens the possibility to investigate self-organizing sensorimotor structures and adaptive autonomy in more depth in the future.

## Data Availability Statement

The raw data supporting the conclusions of this article will be made available by the authors, without undue reservation.

## Author Contributions

FW and ME contributed to the writing of the manuscript. FW developed code and collected results. Both authors contributed to the article and approved the submitted version.

## Funding

FW's contribution was funded by the University of Auckland Doctoral Scholarship program. OAP fees were funded by the School of Computer Science, University of Auckland.

## Conflict of Interest

The authors declare that the research was conducted in the absence of any commercial or financial relationships that could be construed as a potential conflict of interest.

## Publisher's Note

All claims expressed in this article are solely those of the authors and do not necessarily represent those of their affiliated organizations, or those of the publisher, the editors and the reviewers. Any product that may be evaluated in this article, or claim that may be made by its manufacturer, is not guaranteed or endorsed by the publisher.
